# Supplementary material for: Few-layer bismuth selenide cathode for low-temperature quasi-solid-state aqueous zinc metal batteries
Source: Nat Commun. 2022 Feb 8;13:752. doi: 10.1038/s41467-022-28380-y (PMC8825835; doi:10.1038/s41467-022-28380-y)
Supplement: Supplementary file 1 — Supplementary Information [file 41467_2022_28380_MOESM1_ESM.pdf]

SUPPLEMENTARY INFORMATION

Few-layer bismuth selenide cathode for low-temperature quasi-solid-state aqueous zinc metal batteries

Yuwei Zhao[1], Yue Lu[2], Huiping Li[3], Yongbin Zhu[4], You Meng[1], Na Li[1], Donghong Wang[1], Feng Jiang[4], Funian Mo[1], Changbai Long[5], Ying Guo[1], Xinliang Li[1], Zhaodong Huang[1], Qing Li,[1] Johnny C. Ho[1], Jun Fan[1], Manling Sui[2], Furong Chen[1], Wenguang Zhu[3*], Weishu Liu[4*], and Chunyi Zhi[1,6*]

[1]Department of Materials Science and Engineering, City University of Hong Kong, Hong Kong, China.

[2]Institute of Microstructure and Properties of Advanced Materials, Beijing University of Technology, Beijing, China.

[3]International Center for Quantum Design of Functional Materials (ICQD), Hefei National Laboratory for Physical Sciences at the Microscale, Department of Physics, University of Science and Technology of China, Hefei, China.

[4]Department of Materials Science and Engineering, Southern University of Science and Technology, Shenzhen, China.

[5]School of Advanced Materials and Nanotechnology, Xidian University, Xi'an, China.

[6]Centre for Functional Photonics, City University of Hong Kong, Kowloon, Hong Kong

*e-mail: wgzhu@ustc.edu.cn; liuws@sustech.edu.cn; cy.zhi@cityu.edu.hk

Supplementary Figure 1 Schematic illustration of the preparation of E-Bi_2Se_3.

Supplementary Figure 2 Schematically rhombohedral crystal structure of the prepared E-Bi_2Se_3.

Supplementary Figure 3 Rietveld refinement of the XRD pattern of P-Bi_2Se_3

Supplementary Figure 4 TEM image of P-Bi_2Se_3 and corresponding SAED pattern.

Supplementary Figure 5 ADF-STEM image, EDS, Li K-edge EELS and HAADF-STEM image of E-Bi_2Se_3.

Supplementary Figure 6 (a) XPS survey spectra of the as-prepared P-Bi_2Se_3 (upper) and exfoliated E-Bi_2Se_3 (lower). (b) Thermogravimetric analysis (TGA) curves of P-Bi_2Se_3 and E-Bi_2Se_3.

Supplementary Figure 7 XPS spectra of Se 3*d* signals for E-Bi_2Se_3 before and after wash.

Supplementary Figure 8 Overview of E-Bi_2Se_3 nanosheets and corresponding histogram of E-Bi_2Se_3 thickness.

Supplementary Figure 9 Preparation and characterization of HC-EGPAM hydrogel.

Supplementary Figure 10 The working mechanism of the Zn||P-Bi_2Se_3 cell.

Supplementary Figure 11 Rate capability and cycling stability of the quasi-solid Zn||E-Bi_2Se_3 at -50 - 50 °C.

Supplementary Figure 12 Comparison of discharge volumetric capacity of E-Bi_2Se_3 and P-Bi_2Se_3.

Supplementary Figure 13 The quantum spin Hall effect of P-Bi_2Se_3 and E-Bi_2Se_3.

Supplementary Figure 14 Zn||E-Bi_2Se_3 cells with different cathode mass loadings.

Supplementary Figure 15 Demonstration of the flexible Zn||E-Bi_2Se_3 cell.

Supplementary Figure 16 Discharge curves of Zn||E-Bi_2Se_3 cell connected to an low power direct current-direct current boost converter. Ragone plot of Zn||E-Bi_2Se_3 cell.

Supplementary Figure 17 Electrochemical performance of the Zn||E-Bi_2Se_3 cells in aqueous electrolyte.

Supplementary Figure 18 Magnified XRD patterns from Figure 5a.

Supplementary Figure 19 *Ex situ* Raman spectra of the E-Bi_2Se_3 cathode.

Supplementary Figure 20 SAED patterns of E-Bi2Se3 at fully discharged state.

Supplementary Figure 21 TEM image and high-resolution TEM image of E-Bi_2Se_3 at a full discharged state.

Supplementary Figure 22 ADF-STEM, HAADF-STEM image, EELS mappings and EDS plot of $Zn_xBi_2Se_3$.

Supplementary Figure 23 Corresponding TEM-EDS spectrum of fully discharged E-Bi_2Se_3.

Supplementary Figure 24 SEM image of the E-Bi_2Se_3 electrode after 3 cycles.

Supplementary Figure 25 High-resolution Zn 2*p* core level spectra of E-Bi_2Se_3 at selected stages.

Supplementary Figure 26 Schematic diagram of the Zn||E-Bi_2Se_3 full cell.

Supplementary Figure 27 EIS plots and fitted spectra of P-Bi_2Se_3 and E-Bi_2Se_3 cells at varying temperatures.

Supplementary Figure 28 The discharge Galvanostatic intermittent titration technique profiles of Zn||E-Bi_2Se_3.

Supplementary Figure 29 The temperature dependent electrical transport experiment. Two two-way highways beneficial from topological protection based on Kramers theorem.

Supplementary Figure 30 Illustrations of the atomic structure and SOC band structures of the 6-QL E-Bi_2Se_3 slab model and the case of charge neutral Zn atoms intercalated in E-Bi_2Se_3 interlayers (ZnxBi_2Se_3).

Supplementary Figure 31 Structural information of pure E-Bi_2Se_3, Zn^{2+} intercalated in three interlayers and neutral Zn atoms intercalated in E-Bi_2Se_3.

Supplementary Figure 32 Schematic drawing of the $Zn_xBi_2Se_3$ with enhanced metal-like conductivity coming from trivial metal surface state.

Supplementary Table 1 ICP-AES and ICP-MS results for prepared E-Bi_2Se_3

Supplementary Table 2 Crystal data and structure refinement conditions for the P-Bi_2Se_3 and E-Bi_2Se_3

Supplementary Table 3 Fitted impendence parameters of Zn||P-Bi_2Se_3 and Zn||E-Bi_2Se_3 cells.

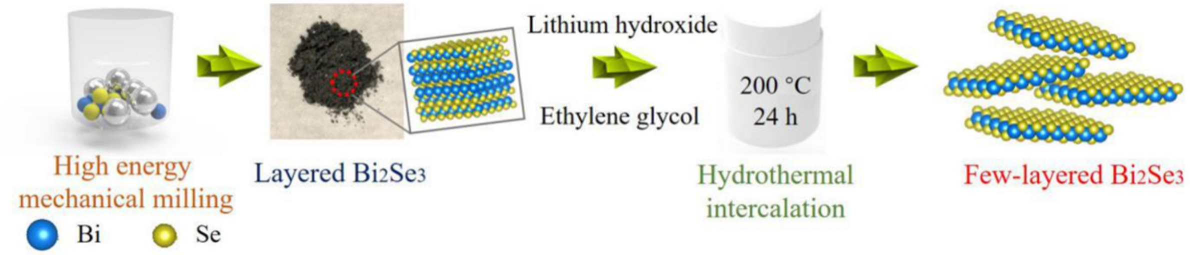

Supplementary Figure 1 Schematic illustration of the preparation of a few layer Bi_2Se_3 nanosheets (E-Bi_2Se_3) by a high energy mechanical milling and hydrothermal intercalation approach. The specific process during exfoliation follows the chemical formulas[1]:

$$Bi_2Se_3 + xLiOH \rightarrow Li_x^0Bi_2Se_3 + xOH^- \qquad (1)$$

$$2Li_x^0Bi_2Se_3 + xH_2O \rightarrow 2Bi_2Se_3 + 2xLiOH + xH_2 \quad (2)$$

Ethylene glycol as solvent is essential for the intercalation process in the hydrothermal reaction. Water is an important cleaning solvent to remove Li^+ as rapid combination of Li^+ and water to form LiOH hydrate. It is easy to eliminate organic ethylene glycol and LiOH by rinsing with acetone.

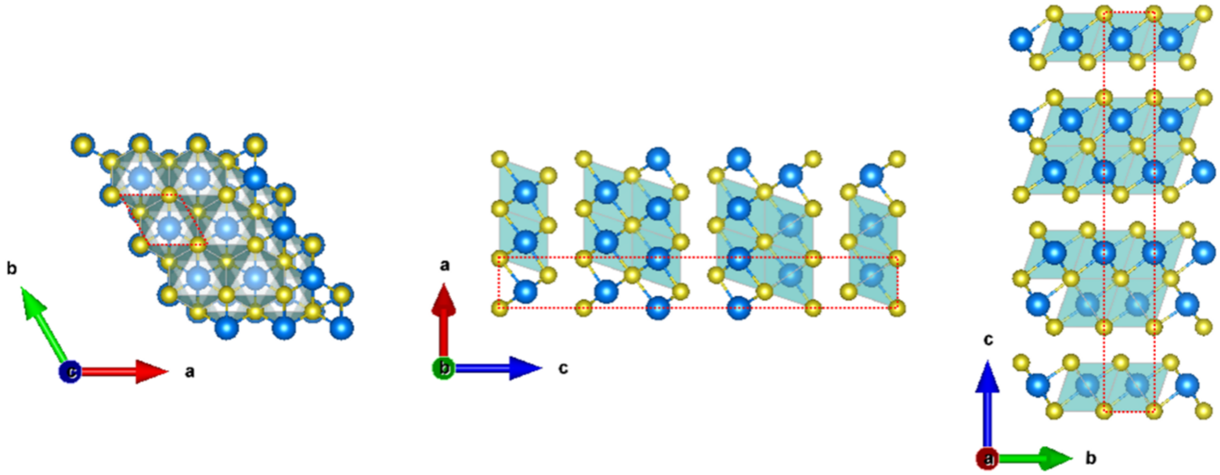

Supplementary Figure 2 Schematically rhombohedral crystal structure of the prepared E-Bi_2Se_3 in the directions of (a) [001], (b) [010] and (c) [100] (red dotted lines show single unit cell). The crystal structure of Bi_2Se_3 is formed by a periodic layer composed of quintuple layers (QLs, namely five atomic planes: Se1-Bi-Se2-Bi-Se1) aligned perpendicular to the trigonal c-axis.

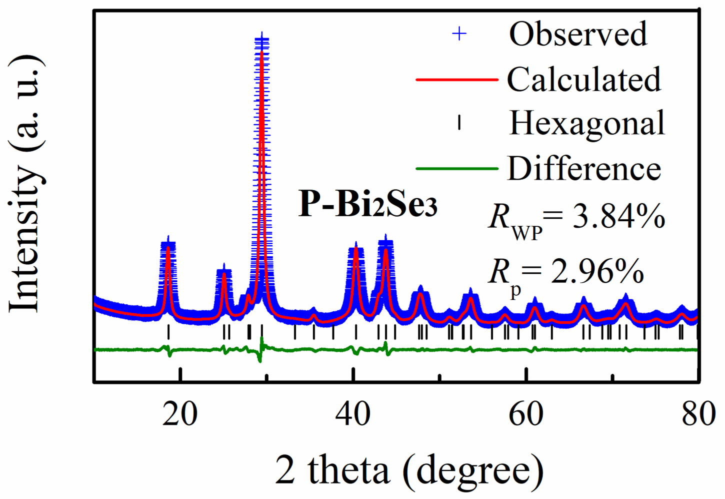

Supplementary Figure 3 Rietveld refinement of the XRD pattern of P-Bi_2Se_3 with reliability factors R_{wp} and R_p of 3.84% and 2.96%, respectively.

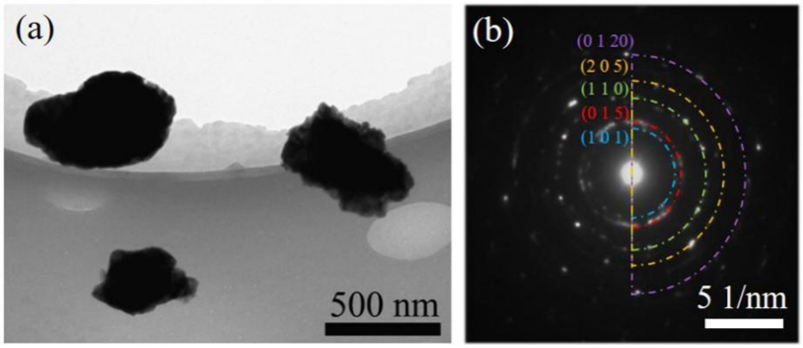

Supplementary Figure 4 (a) TEM image of P-Bi_2Se_3 and (b) corresponding SAED pattern.

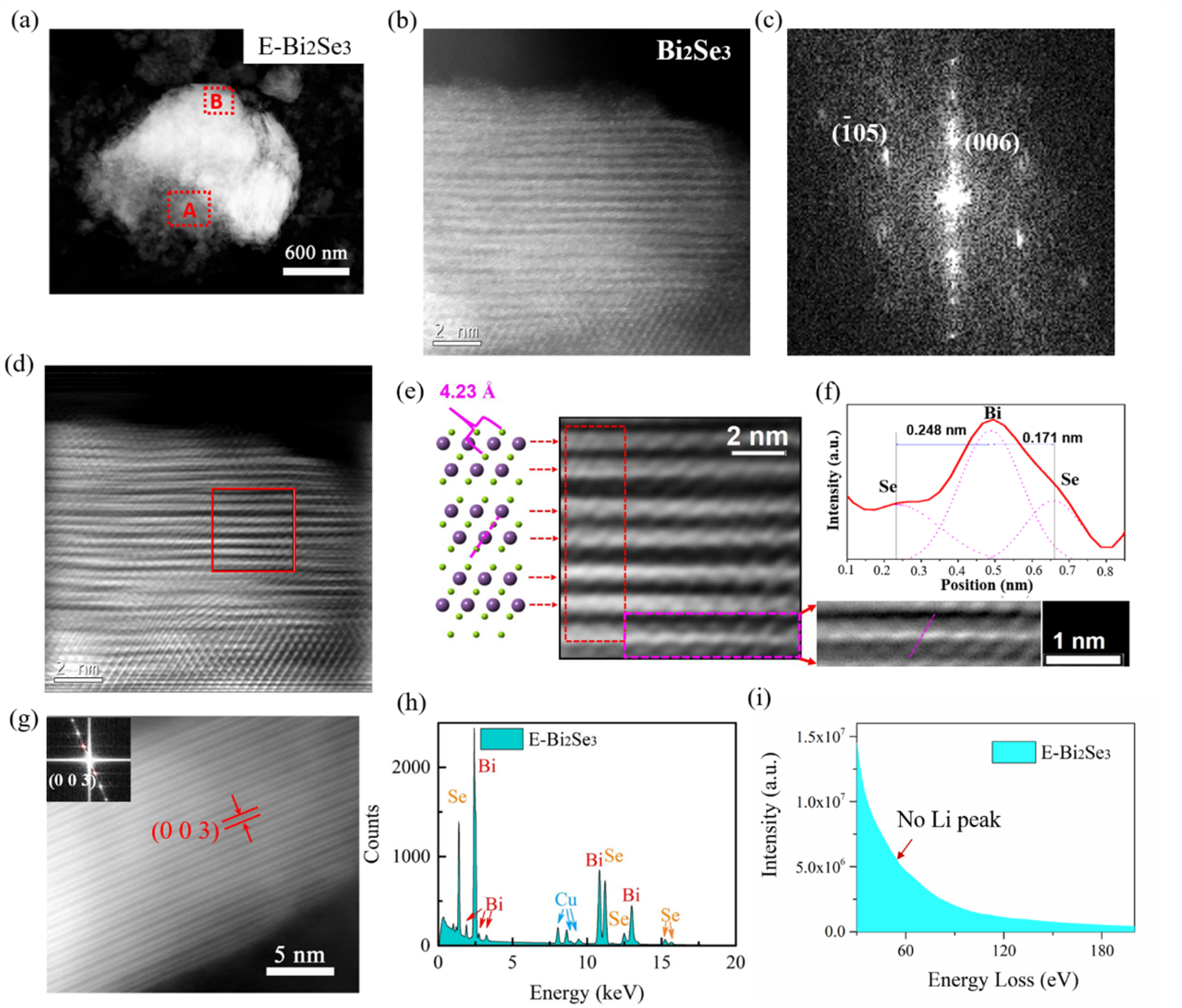

Supplementary Figure 5 (a) Annular dark field-scanning transmission electron microscopy (ADF-STEM) image of E-Bi_2Se_3. The rectangular areas marked A and B are positions where HAADF-STEM images (Figure 1e and Supplementary Figure 5g) are acquired from. (b) HAADF-STEM image of E-Bi_2Se_3 and (c) its corresponding SAED pattern. (d) High-resolution HAADF-STEM image of E-Bi_2Se_3 in (b). (e) Schematic of the atomic configuration of Bi and Se atom alont [010] projection, the green sphere presents the Se atom and purple sphere indicates the Bi atom. The distance of two Se atoms as indicated is 4.23 Å while the spacing of two Se is 4.17Å. Such atomic configuration fits well with the enlarged HAADF image. (f) Average line intensity profile

along the HAADF image (the pink dash line at the bottom of HAADF image), which is cut up from the bottom area of HAADF image in (e). (g) The high-resolution HAADF-STEM image of E-Bi_2Se_3 from the B region in (a). The inset shows corresponding SAED pattern. (h) Energy-dispersive X-ray spectroscopy (EDS) of E-Bi_2Se_3. (i) The electron energy loss spectrum of E-Bi_2Se_3 shows that E-Bi_2Se_3 does not contain Li as there is no peak at 55 eV.

From HAADF images (Supplementary Figure 5b-e) we could identify the atomic configuration of E-Bi_2Se_3. In ideal conditions, the distance between Se-Bi-Se columns is 4.23 Å, and Bi atom sites symmetrically between two Se atoms (the Schematic in Supplementary Figure 5e). We averaged several plots across Se-Bi-Se cluster along pink dashed lines. Assume the atom is a Gaussian shape. The averaged plot (average of 8 line profiles) is fitted with Gaussians. The spacing between two Se-Se along the dash line from fitted Gaussians is 4.14Å with the experimental error of 0.3 Å. Therefore, the confirmation of existence of Se-Bi-Se is reasonably in good confidence.

However, although the HAADF image (Supplementary Figure 5e) does not clearly reveal the atomic configuration, as fitting the peak intensity of line profile in Supplementary Figure 5f, we can still see two Se atoms do not distribute symmetrically around Bi site (the distance of Se-Bi at the left side is 0.248 nm, and the distance of Bi-Se at the right side is 0.171 nm, which is an obvious difference as considering the experimental error of 0.03 nm). Normally, ball-milling, and hydrothermal stripping could affect the crystallinity greatly during the sample preparation. Especially, it is well-known that the electron beam knock-on may displace the atoms in crystal.[2] When we tried to capture the high resolution HAADF image along [010] zone axis, the atomic configuration at different areas may have certain distortion. This can explain the observed unsymmetrical Bi atom sitting between two Se atoms.

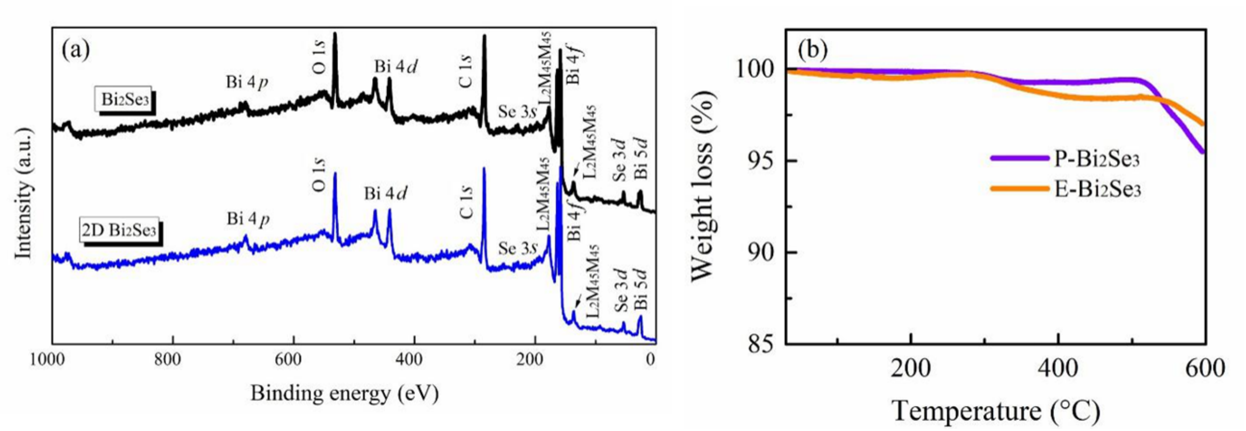

Supplementary Figure 6 (a) XPS survey spectra of the as-prepared P-Bi_2Se_3 (upper) and exfoliated E-Bi_2Se_3 (lower). (b) Thermogravimetric analysis (TGA) curves of P-Bi_2Se_3 and E-Bi_2Se_3.

XPS test also reveals that the Li/Bi/Se atomic ratio in E-Bi_2Se_3 sample is 0.00:1.00:1.45, respectively. TGA is performed under N_2 atmosphere at a linear heating rate of 10 °C min^{-1} to test whether there is any liquid intercalated into the gap. As shown in Supplementary Figure 6b, weight loss of Bi_2Se_3 starts from 300 ° C ascribed to the loss of Se. There is no liquid intercalated into the interlayer of E-Bi_2Se_3.

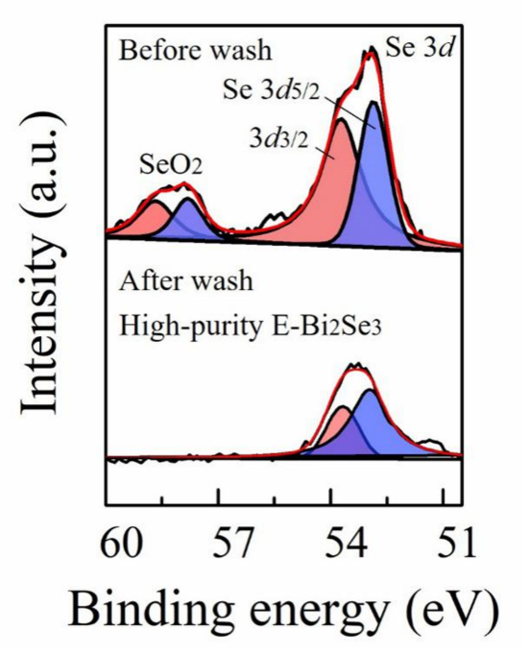

Supplementary Figure 7 XPS spectra of Se 3*d* signals for E-Bi_2Se_3 before (upper) and after wash (lower) with tetramethylammonium hydroxide (TMAH)/NaOH/NaCl aqueous solution and DI water. The small amount of SeO_2 impurity can be fully removed and high-purity E-Bi_2Se_3 can be obtained.

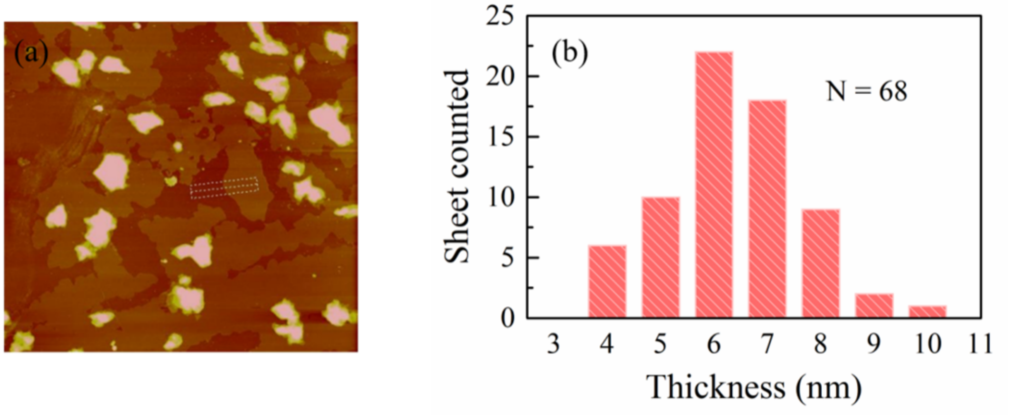

Supplementary Figure 8 Overview of E-Bi_2Se_3 nanosheets (NSs) on a substrate area of ~100 μm^2 (a) and corresponding histogram of E-Bi_2Se_3 thickness (b) N indicates the number of the NSs.

NSs with average thickness around 6 QLs are major products, and with heights about ＜4 or ＞10 QLs are minority.

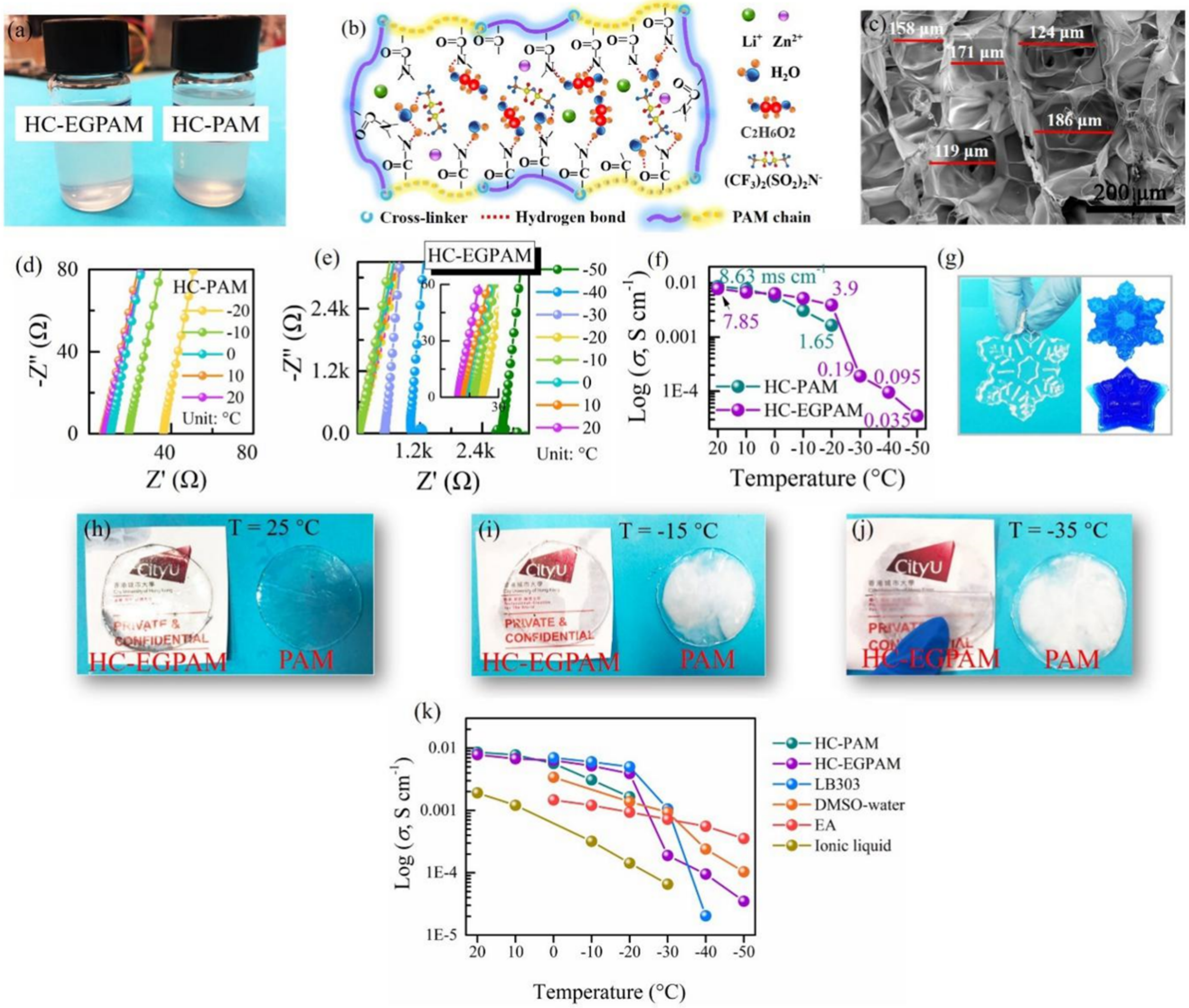

Supplementary Figure 9 Preparation and characterization of HC-EGPAM hydrogel electrolyte for assembly of the quasi-solid cells. (a) A photograph of the transparent dispersion of HC-EGPAM and HC-PAM electrolytes before the in-situ free-radical polymerization. (b) Schematic illustration of the hydrogen interactions among high concentration of salts (HC), ethylene glycol (EG), polyacrylamide (PAM) and water in the HC-EGPAM gel electrolyte. (c) SEM image of the cross-section of the PAM hydrogel after freeze drying. AC impedance spectra of the HC-PAM (d)

and HC-EGPAM (e) hydrogel electrolyte at different temperatures, and the calculated ionic conductivity of HC-EGPAM and HC-PAM (f). The inset numbers in (f) are calculated ionic conductivities of HC-EGPAM at lower temperatures. (g) Optical images of the transparent HC-EGPAM which can be processed into various shapes and dyed blue for visibility. Photographs of the as-prepared HC-EGPAM hydrogel with a typical PAM gel as control at 25 °C (h), -15 °C (i) and -35 °C (j) demonstrating the superior freeze-resistant properties. (k) Comparison of the ionic conductivity of HC-EGPAM and HC-PAM with various other low-temperature electrolytes.[3-6] EA is shorted for ethyl acetate-based cosolvent electrolyte, and carbonate - based electrolyte is denoted as LB303.

Acrylamide (AM) monomers are polymerized together assisted by ammonium persulfate as the initiator and bisacrylamide as the cross-linker within the highly concentrated salts (HC) and ethylene glycol (EG) forming a skeleton by covalent cross-linking bonds and physical hydrogen bonds (Supplementary Figure 9b). The SEM image of the cross-section of the freeze-dried PAM hydrogel exhibits amounts of uniform porous channels with 150 ± 30 μm in diameter (Supplementary Figure 9c), which benefits the water-retention and free movement of electrolyte ions endowing a superior ionic conductivity. From Supplementary Figure 9d and 9e, we obtain the ionic conductivity of the HC-EGPAM and HC-PAM hydrogels from their AC impedance spectra demonstrating that the ohmic resistance for HC-EGPAM with varying temperatures shows a mild increase from 9.38 to 19.05 Ω (increase from 2.9 to 36 Ω for HC-PAM). Notably, HC-EGPAM exhibits a higher ohmic resistance (9.38 Ω) than that of HC-PAM (2.9 Ω) at room temperature (RT) suggesting disadvantageous ion transport within the 3D skeleton mainly due to a higher cross-linking degree of HC-EGPAM. An obvious contrast exists between the ionic conductivity of HC-EGPAM and HC-PAM hydrogels as shown in Supplementary Figure 9f indicating that the

EG additive actually contributes to the anti-freezing behavior of the hydrogel, and further renders the electrolytes with better ionic conductivity in cold weather. The as-made HC-EGPAM hydrogels are transparent, easily processed into various morphologies (Supplementary Figure 9g). Moreover, the usual PAM hydrogel easily freezes at sub-zero temperature while HC-EGPAM hydrogel displays high adhesiveness, superior freezing tolerance, and excellent flexibility even when exposed at a low temperature of -35 °C (Supplementary Figure 9h, i and j). The HC-EGPAM hydrogel electrolyte shows superior ionic conductivity comparable to DMSO-water electrolyte,[3] ethyl acetate (EA)-based cosolvent electrolyte,[4] carbonate - based electrolyte (LB303),[5] and ionic liquid electrolyte (Supplementary Figure 9k).[6]

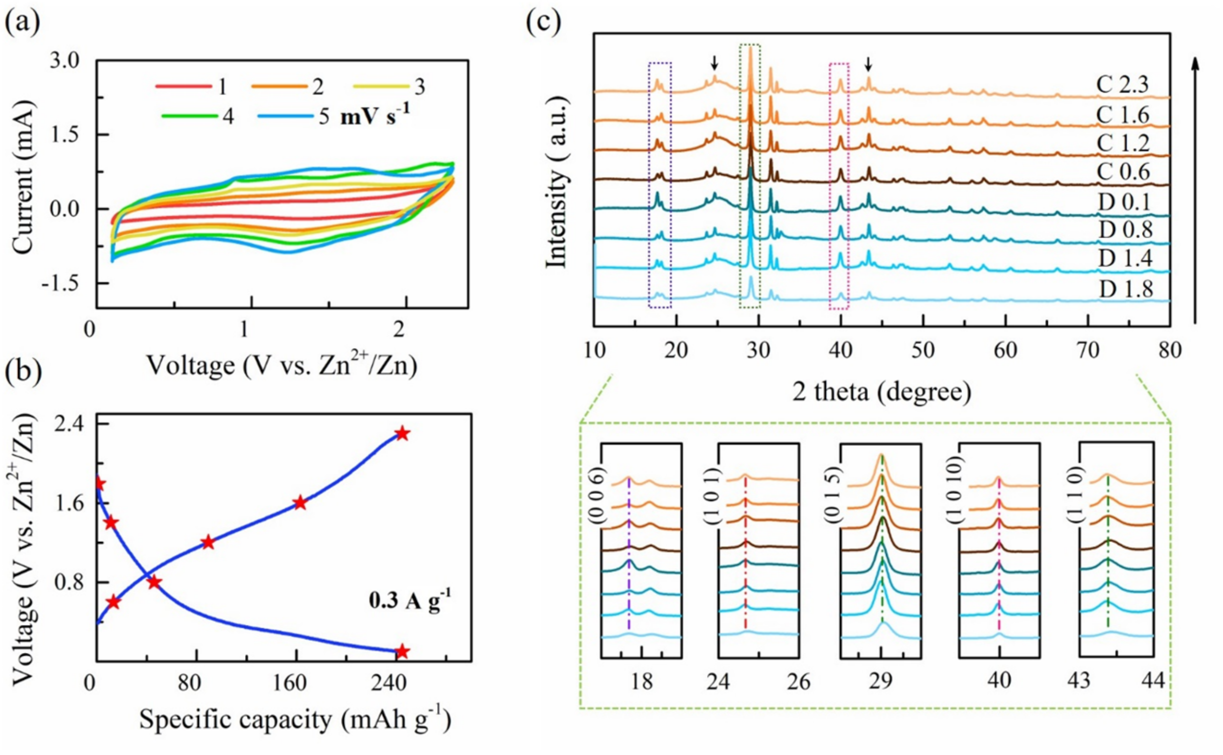

Supplementary Figure 10 The working mechanism of the P-Bi_2Se_3 cathode in 1m $Zn(TFSI)_2$ and 21m LiTFSI/H_2O where m is molality (mol kg^{-1}) aqueous electrolyte at room temperature. (a) CVs of Zn||P-Bi_2Se_3 cells in the liquid electrolyte at different scan rate. (b) GCD profiles for the third

cycle of the AZIB at 0.3 A g^{-1}, in which the red star refers to the positions at which the XRD patterns are obtained. (c) Evolution of *ex situ* XRD patterns during the charge/discharge process for P-Bi_2Se_3 at different states corresponding to (b) where red curves are corresponding to the discharge process while the blue corresponding to the charge process.

CVs of Zn||P-Bi_2Se_3 cells display no significant redox peaks in Supplementary Figure 10a. GCD profiles for the third cycle also show no obvious charge and discharge platforms (Supplementary Figure 10b). The characteristic peaks of P-Bi_2Se_3 cathodes remain unchanged at the selected states different from those of E-Bi_2Se_3 showing that the amount of Zn^{2+} embedded is limited and the main capacity contribution is from capacitance contribution (Supplementary Figure 10c).

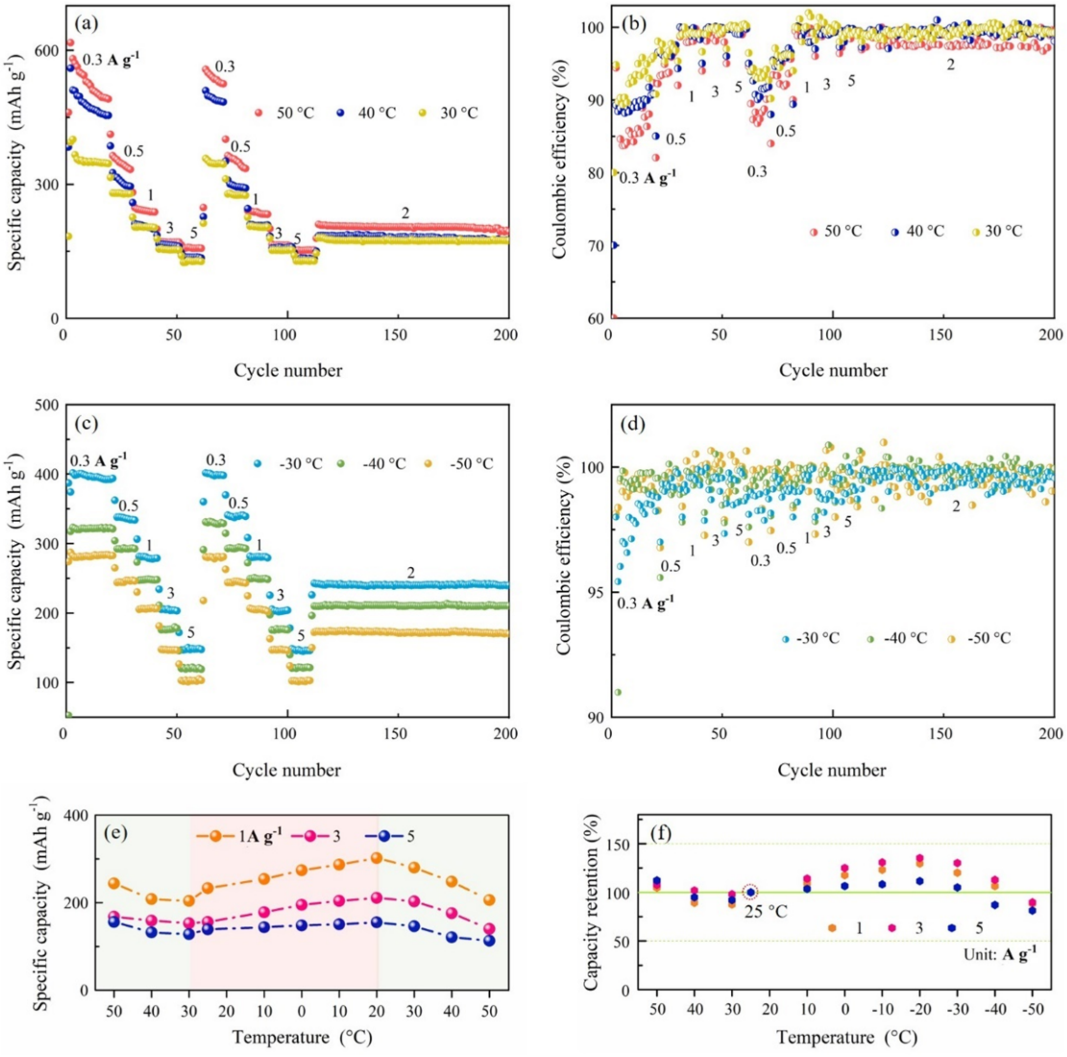

Supplementary Figure 11 Rate capability and cycling stability of the quasi-solid Zn||E-Bi_2Se_3 at (a, b) 30 - 50 °C, and (c, d) -30 - -50 °C. (e) Discharge specific capacity versus temperature of Zn||E-Bi_2Se_3 cells at various current rates and a wide temperature range of -50 °C - 50 °C. The red area represents better cell performance at lower temperature. The green area represents worse cell performance at lower temperature. (f) Capacity retention of Zn||E-Bi_2Se_3 cells at various temperatures and current rates. Here the capacity retention is calculated by $\left(\frac{\text{discharge capacity at other temperature}}{\text{discharge capacity at 25 °C}} \times 100\ \%\right)$.

In Supplementary Figure 11a, the Zn||E-Bi_2Se_3 cells deliver discharge capacities of 543, 334, 244, 168, and 156 mAh g^{-1} at specific currents between 0.3 to 5 A g^{-1} at 50 °C. While at 2 A g^{-1}, discharge capacities of 196 mA h g^{-1} can be obtained after 200 cycles. Coulombic efficiency (CE) lower than 100 % at 0.3 and 0.5 A g^{-1} is due to side reactions (Supplementary Figure 11b). As the temperature increases, the chemical reaction intensifies and the side reaction increases. [7]

In Supplementary Figure 11c the Zn||E-Bi_2Se_3 cells deliver discharge capacities of 280, 245, 206, 140 and 113 mAh g^{-1} at specific currents from 0.3 to 5 Ag^{-1} at -50 °C. While at 2 A g^{-1}, discharge capacities of 171 mAh g^{-1} can be obtained after 200 cycles. CE approaching 100% can be achieved in all cycles (Supplementary Figure 11d).

Supplementary Figure 11e summarizes temperature-dependent discharge capacity of Zn||E-Bi_2Se_3 cells at various current rates. The red area shows that the lower the temperature, the better the performance, while the green area indicates that the lower the temperature, the worse the performance of the cell. For the temperature range from -20 °C to -50 °C, the degradation of cell performance is mainly caused by the degradation of electrolyte property as the ionic conductivity may remarkably decrease with the declined temperature in a highly concentrated salt solution. For the temperature range from 30 °C to 50 °C, the better electrolyte property is the main reason that leads to better cell performance as the electronic conductivity of the electrode increases with the decrease of temperature. For the abnormal region from -20 °C to 30 °C, the contribution of E-Bi_2Se_3 electrode to the cell performance is greater than that of the electrolyte.

Supplementary Figure 11f summarizes capacity retention of Zn||E-Bi_2Se_3 cells at various temperatures and current rates (compared to the corresponding discharge capacities at 25 °C). Even at -40 °C, the capacity retentions at 1 and 3 A g^{-1} remain to 106 % and 113 %. At -50 °C, the

capacity retentions of Zn||E-Bi_2Se_3 cells are record-high 88 % (1 A g^{-1}), 90 % (3 A g^{-1}) and 81 % (5 g^{-1}), respectively.

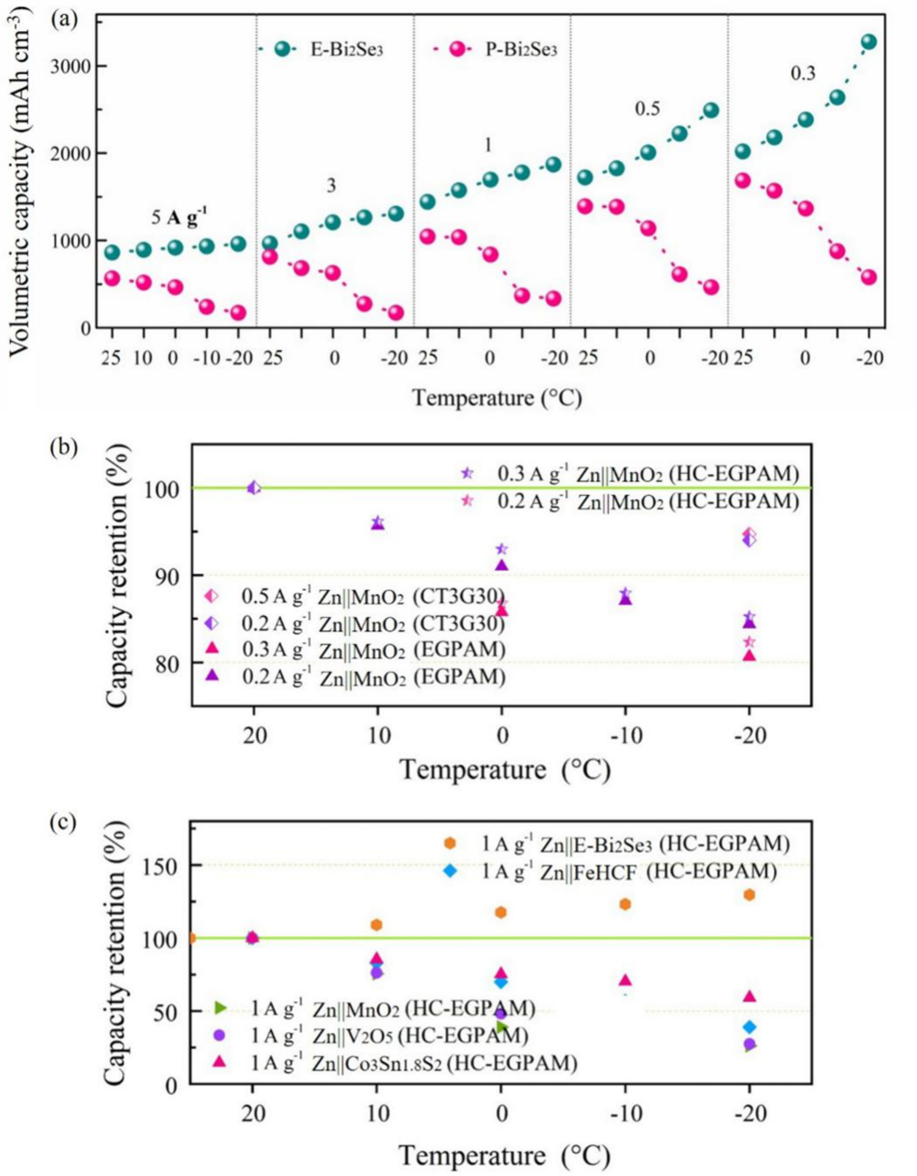

Supplementary Figure 12 (a) Comparison of discharge volumetric capacity at different rates of E-Bi_2Se_3 and P-Bi_2Se_3 cathode (active materials only). (b) Temperature-capacity retention dependence of Zn||MnO_2 cells using HC-EGPAM of this work with other Zn||MnO_2 cells reported using other antifreeze hydrogel electrolytes.[8,9] Here an antifreeze polyacrylamide (PAM) hydrogel

electrolyte with highly concentrated salts incorporated along with ethylene glycol is denoted as HC-EGPAM. PAM hydrogel electrolyte with 2M $ZnSO_4$ salt incorporated along with ethylene glycol is denoted as EGPAM. CT3G30 hydrogel electrolyte uses cotton as the raw material, tetraethyl orthosilicate as the crosslinker, and glycerol as the antifreezing agent, where C, T, 3, G, and 30 represent cellulose, tetraethyl orthosilicate (TEOS), the milliliters of TEOS added, glycerol, and the milliliters of glycerol added, respectively. (c) Temperature-capacity retention dependence of E-Bi_2Se_3 and other cathodes using the same HC-EGPAM electrolyte.

Although E-Bi_2Se_3 has a larger volume, E-Bi_2Se_3 still exhibits the superiority on volumetric capacity in comparison with P-Bi_2Se_3. The volumetric capacities are calculated based on the mass and density of the E-Bi_2Se_3 and P-Bi_2Se_3 active materials.

To rule out the contribution from the electrolyte, we compare Zn||MnO2 cells using HC-EGPAM of this work with other antifreeze hydrogel electrolytes already reported (Supplementary Figure 12b).[8,9] Capacities of Zn||MnO2 cells using HC-EGPAM and other antifreeze hydrogel electrolytes all decrease with decreasing temperature, and Zn||MnO2 cells using HC-EGPAM and EGPAM electrolytes show similar capacity retention rates at different temperatures. This phenomenon indicates the abnormal low-temperature performance doesn't originate from the electrolyte we used.

Then, we also compare our topological insulator E-Bi_2Se_3 electrode with Prussian blue analogue (FeHCF), V_2O_5, MnO_2 and $Co_3Sn_{1.8}S_2$ electrodes in the same HC-EGPAM (Supplementary Figure 12c). Considering that the performance of other electrode materials remarkably decreases with the decrease of temperature, we confirm that the exfoliation-enhanced topological surface state of the E-Bi_2Se_3 is the key to achieve the improved performance at low temperature. Of course, to obtain

superior low-temperature performance, it is essential to ensure that the electrolyte is in a good working condition at low temperature.

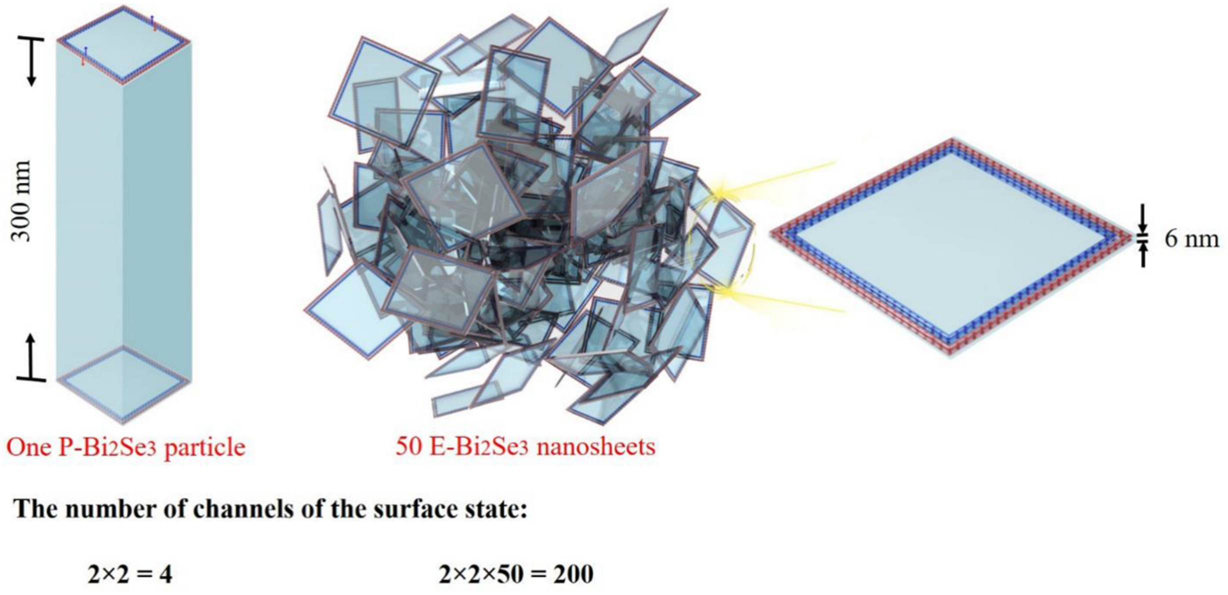

Supplementary Figure 13 The quantum spin Hall effect (QSHE) of P-Bi_2Se_3 and E-Bi_2Se_3. The surface state has two channels with opposite spin chirality. Exfoliating P-Bi_2Se_3 into E-Bi_2Se_3 greatly increases the number of channels of the surface state from 4 to 200 in this example. The short arrows in blue and red represent the direction of spin.

After exfoliation, the reduced concentration of bulk carriers in E-Bi_2Se_3 highlights the contribution of conductivity from surface topological states, significantly increasing electronic conductivity and boosting the electron transfer kinetics.[10,11]

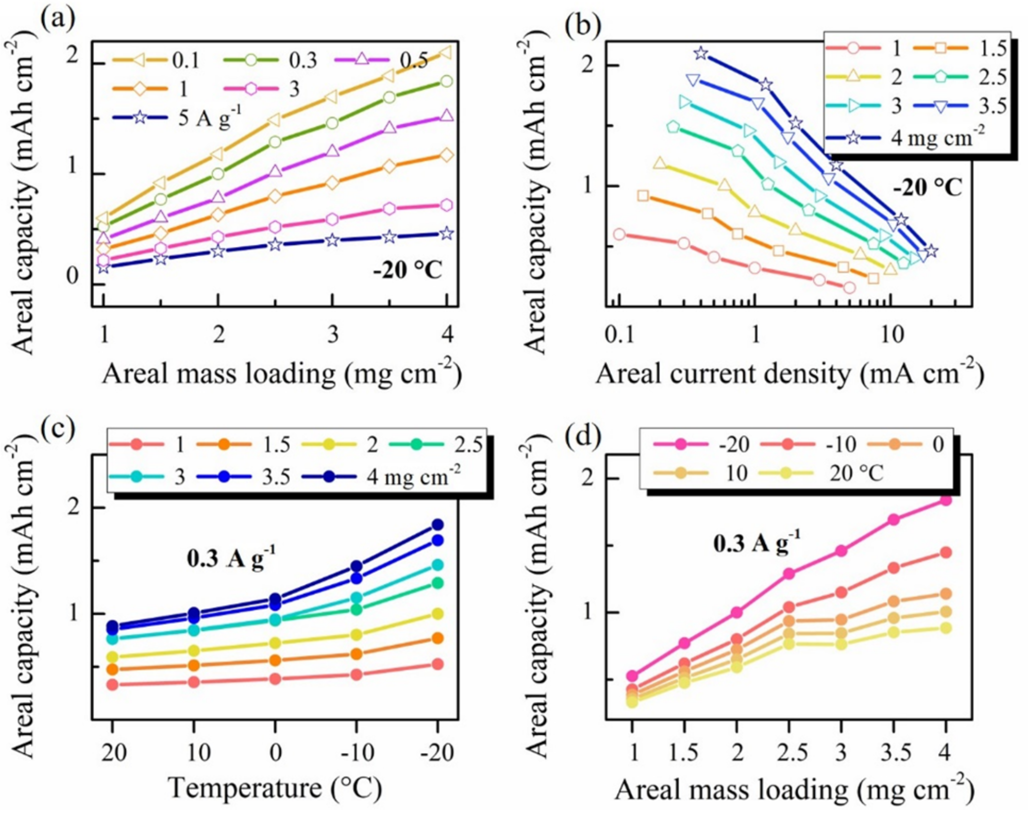

Supplementary Figure 14 Zn||E-Bi_2Se_3 cells with different cathode mass loadings. (a) The areal capacity of E-Bi_2Se_3 cathodes with mass loading of 1-4 mg cm^{-2} measured at specific currents from 0.1 A g^{-1} to 5 A g^{-1} at -20 °C. (b) The areal capacity vs. the areal specific current of E-Bi_2Se_3 cathodes with a mass loading of 1-4 mg cm^{-2} at -20 °C, respectively. (c) Temperature-dependent areal capacity of E-Bi_2Se_3 cathodes with mass loading of 1-4 mg cm^{-2} at 0.3 A g^{-1}. (d) The areal capacity of E-Bi_2Se_3 cathodes with mass loading of 1-4 mg cm^{-2} from -20 °C to 20 °C at 0.3 A g^{-1}.

To evaluate utility of Zn||E-Bi_2Se_3 cells, E-Bi_2Se_3 cathodes with a mass loading of 1-4 mg cm^{-2} are tested at specific currents of 0.1 A g^{-1} to 5 A g^{-1}. Supplementary Figure 14a plots the areal capacity vs. the areal mass loading of the electrodes at -20 ° C. The areal capacity increases linearly with the increasing areal mass loading at relatively low specific currents (<3 A g^{-1}). At the specific current >3 A g^{-1}, the dependence slightly deviates from the linear relationship.

Supplementary Figure 14b shows the areal capacity as a function of the areal specific current in which the E-Bi_2Se_3 cathode with a mass loading of 4 mg cm^{-2} exhibits areal capacities of 2.1, 1.84, 1.52, 1.17, 0.72, and 0.46 mAh cm^{-2} at the areal specific current of 0.4, 1.2, 2, 4, 12, and 20 mA cm^{-2}, respectively. Temperature-dependent areal capacity of E-Bi_2Se_3 cathodes and the areal capacity vs. areal mass loading at 0.3 A g^{-1} are displayed in Supplementary Figure 14c and Supplementary Figure 14d, respectively. The E-Bi_2Se_3 cathode with a mass loading of 4 mg cm^{-2} delivers areal capacities of 1.84, 1.45, 1.14, 1.01 and 0.89 mAh cm^{-2} at the temperature of -20, -10, 0, 10, and 20 ° C, respectively.

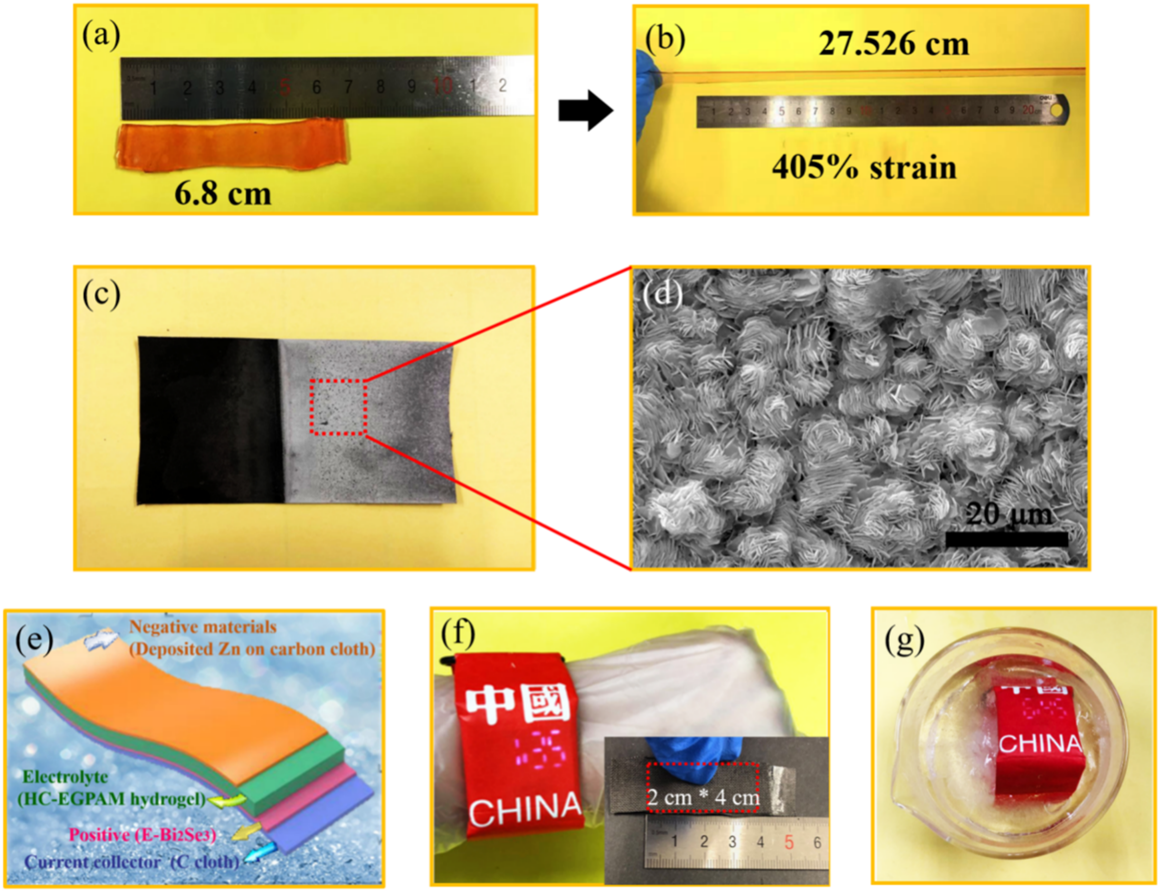

Supplementary Figure 15 The relaxed (a) and elongated (b) states of the HC-EGPAM hydrogel dyed in orange for visibility revealing its excellent stretchability. An optical photo of the flexible

electrodeposited Zn anode on the carbon cloth (c) and the corresponding SEM image (d). (e) Schematic illustration of the quasi-solid AZIB. Demonstration of the two flexible Zn||E-Bi_2Se_3 cell connected in series powering a 3V watch at (f) 25 ° C and (g) 0 ° C.

The HC-EGPAM readily stretched to 405 % strain which is benefited from dynamically breaking and recombining of plenty of inter- and intramolecular hydrogen bonds among the polymer network dissipating energy under deformation.[12]

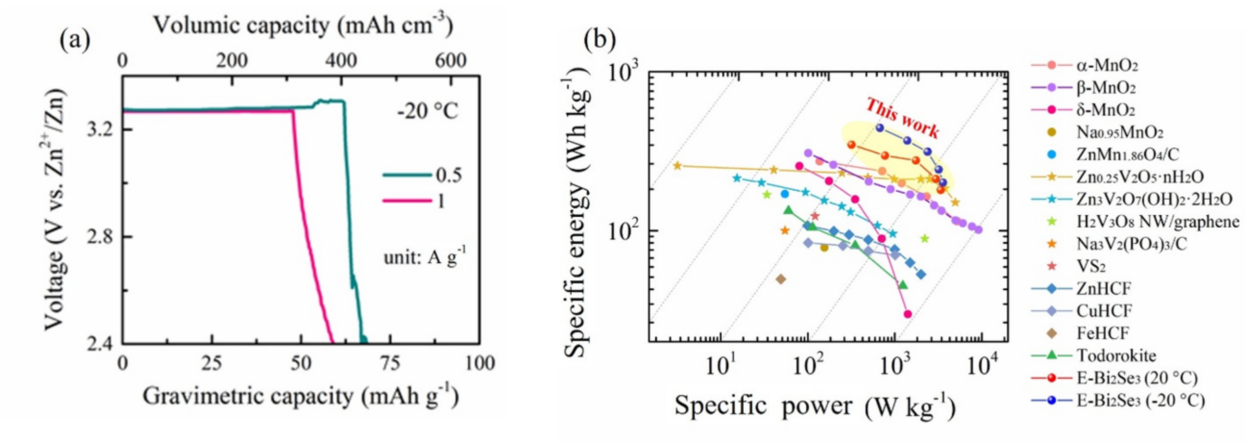

Supplementary Figure 16 (a) Discharge curves of Zn||E-Bi_2Se_3 cell when connected to a low power direct current-direct current (DC-DC) boost converter. (b) Ragone plot of Zn||E-Bi_2Se_3 cell in comparison with other aqueous ZIBs.[13-25] The quasi-solid Zn||E-Bi_2Se_3 cell output a maximum specific energy of 441 Wh kg^{-1} at -20 °C a specific power of 683 W kg^{-1} superior to most representative aqueous cells based on Mn-based oxides, V-based oxides and sulfides and Prussian blue analogues, which is promising for large-scale low-temperature energy storage applications.

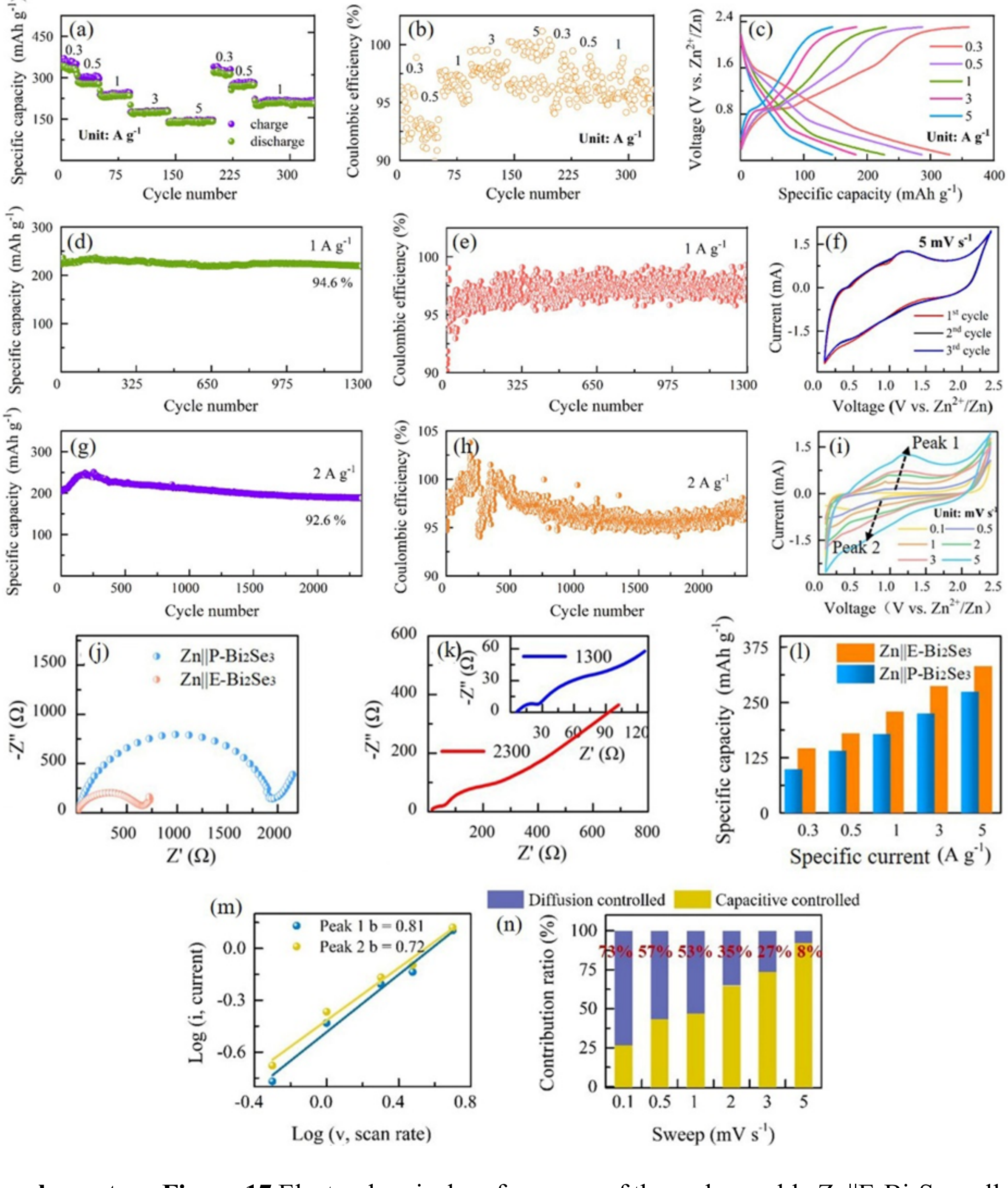

Supplementary Figure 17 Electrochemical performance of the rechargeable Zn||E-Bi_2Se_3 cells in 1m $Zn(TFSI)_2$ and 21m LiTFSI/H_2O aqueous electrolyte at room temperature. Rate capability (a) and the corresponding CE (b) and galvanostatic charge/discharge (GCD) profiles (c) of the AZIB at varying current rates. Cycling stability at 1 A g^{-1} (d, e) and 2 A g^{-1} (g, h). CV profiles for the

first three cycles at 5 mV s^{-1} (f) and at varying current rates (i). (j) Electrochemical impedance spectra (EIS) of uncycled Zn||E-Bi_2Se_3 and Zn||P-Bi_2Se_3 cells. (k) EIS of Zn||E-Bi_2Se_3 cells after 2300 and 1300 cycles at 2 and 1 A g^{-1}, respectively. The fitting results of the above EIS plots are presented in Table S3. (l) The comparison of discharge specific capacity versus specific current of Zn||P-Bi_2Se_3 and Zn||E-Bi_2Se_3 cells in liquid electrolyte. (m) The linear fit between log (i, current) and log (v, scan rate) at cathodic and anodic peaks. (n) Capacitance and battery type contribution ratios.

Discharge capacities of 332, 287, 229, 181, and 146 mAh g^{-1} are recorded at rates of 0.3, 0.5, 1, 3, and 5 A g^{-1}, respectively, and corresponding GCD profiles with discharge platforms around 1.6 and 0.8 V are given in Supplementary Figure 17a, b and c. The capacity loss at the low specific current of 0.3 A g^{-1} with relatively low CE is ascribed to the existing irreversible side reactions (Supplementary Figure 17b).[26] When the specific current is reset to 0.3 A g^{-1}, the capacity is almost completely restored indicating the excellent structural stability of E-Bi_2Se_3. CV curves of the Zn||E-Bi_2Se_3 cell coincide perfectly in the first three cycles at a scan rate of 5 mV s^{-1}, manifesting the highly reversible reaction of the cell (Supplementary Figure 17f). CV measurements at varying rates from 0.1 to 5 mV s^{-1} are also carried out in Supplementary Figure 17i. With the increase of scan rates, the CV profiles exhibit similar outlines and gradually increased currents. Meanwhile, all of them show less obvious redox peaks. Furthermore, the cycle stability of the Zn||E-Bi_2Se_3 cells is tested at 1 A g^{-1} (Supplementary Figure 17d and e) and 2A g^{-1} (Supplementary Figure 17g and h). Memorably, the cell shows superior cycling performance, demonstrating high capacity retentions of 94.6 % over 1300 cycles at 1 A g^{-1} and 92.6 % over 2300 cycles at 2 A g^{-1} with CE approaching 100%. EIS profiles of the original Zn||E-Bi_2Se_3 and Zn||P-Bi_2Se_3 cells are conducted in Supplementary Figure 17j, which can be fitted by equivalent circuit 1 in Table. S3. Impressively,

the original Zn||E-Bi_2Se_3 has much smaller R_s, R_{ct} and Z_W compared to those of Zn||P-Bi_2Se_3 cells. The cycled cells both display two depressed semicircles in the high-frequency region and one line in low-frequency end (Supplementary Figure 17k), indicating the existence of obvious interface component.[27] Fitting the EIS data by equivalent circuit 2 in Table. S3, we found that R_s, R_{ct}, R_i, and Z_W all increase as the cycle goes on, which is likely caused by the structural change of electrodes.[28] In comparison, the Zn||E-Bi_2Se_3 cells show much better capacities achieved at different rates than 274, 225, 178, 141 and 99 mAh g^{-1} at rates of 0.3, 0.5, 1, 3, and 5 A g^{-1} of Zn||P-Bi_2Se_3 cells (Supplementary Figure 17l). Understanding the operative mechanisms of energy storage systems is extremely important. According to Equation (1), the b values of peak 1 and 2 are 0.81 and 0.72 (Supplementary Figure 17m), respectively, demonstrating that presence of a synergistic charge storage process. Both diffusion-controlled and capacitive behaviours in Zn||Bi_2Se_3 cells are responsible for the fast kinetics during the discharge/charge process. To further specify the capacitive contribution at a certain scan rate, the Equation (2) is employed. The result shows that proportions of ≈73-8 % of the whole capacity originate from the cell-type contribution at scan rates of 0.1-5 mV s^{-1}(Supplementary Figure 17n).

$$i = av^b \quad (1)$$

$$i = k_1 v + k_2 v^{1/2} \quad (2)$$

When capacitor behavior is involved, Zn^{2+} is adsorbed on the E-Bi_2Se_3 surface from the electrolyte, where topological surface states of E-Bi_2Se_3 still play the same key role. The surfaces act as conductors because their electrons are subject to strong spin-orbit interaction existing on their electrons. Higher electronic conductivity is still obtained of the topological E-Bi_2Se_3 cathode at

lower temperatures, accelerating the electron transfer kinetics of the cell reactions at lower temperature.

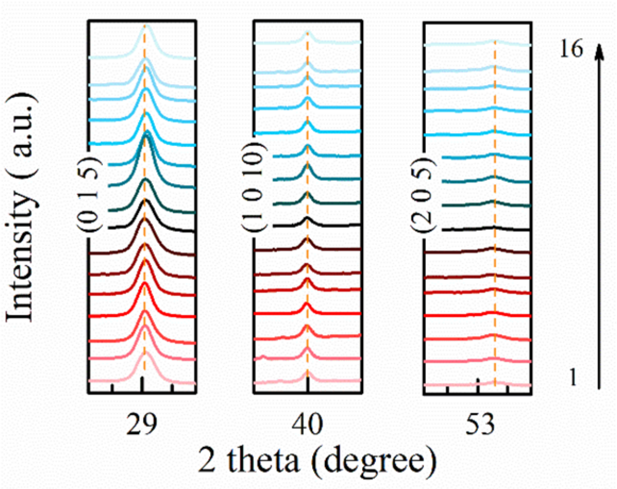

Supplementary Figure 18 Magnified XRD patterns from Figure 5a corresponding to the (0 1 5), (1 0 10) and (2 0 5) planes of E-Bi_2Se_3.

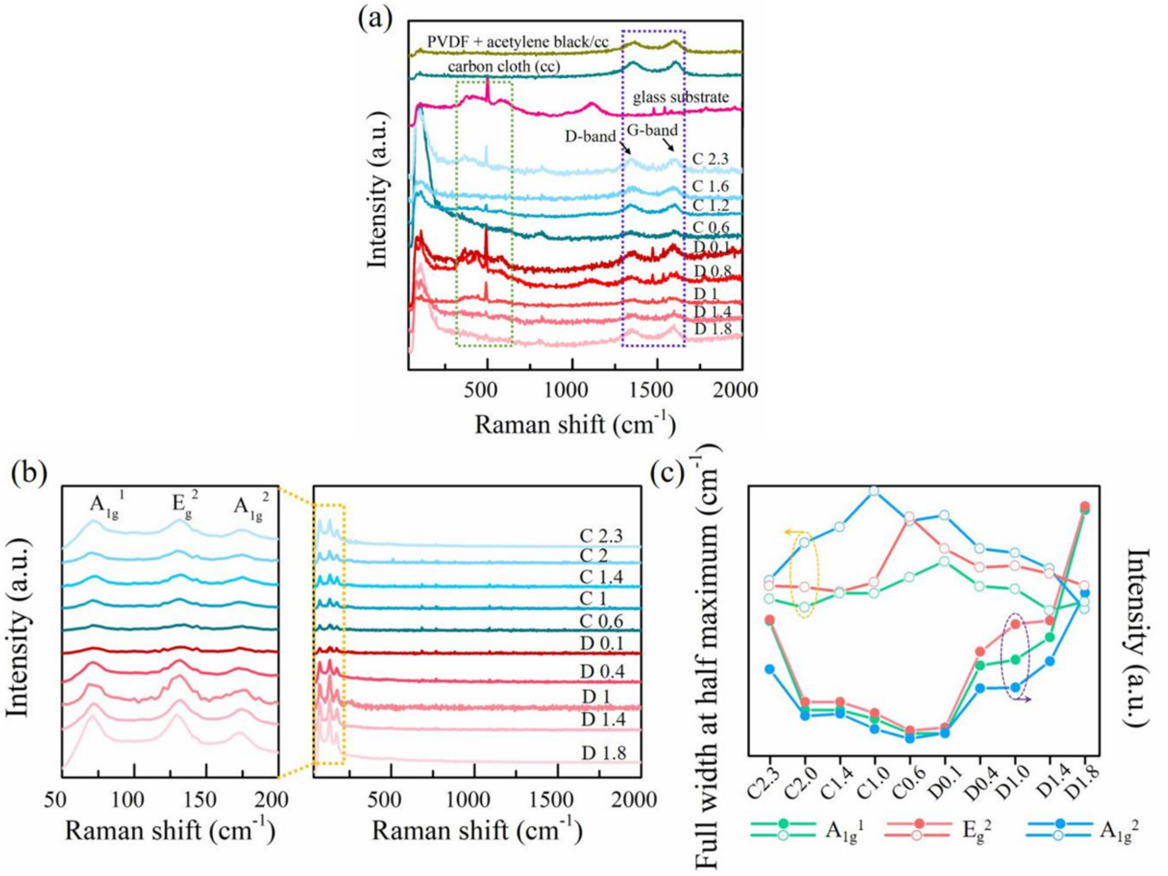

Supplementary Figure 19 (a) *Ex situ* Raman spectra (35-2000 cm^{-1}) of the E-Bi_2Se_3 cathode at the selected states corresponding to the charge/discharge process. (b) *Ex situ* Raman spectra (50-200 cm^{-1} and 35-2000 cm^{-1}) of the recycled E-Bi_2Se_3 obtained after washing of E-Bi_2Se_3 cathode and centrifugal collection at the selected states corresponding to the charge/discharge process in Figure 5a. (c) Intensity and full width at half maximum (FWHM) measurements of Raman-active modes for E-Bi_2Se_3 ($^1A_{1g}$, ^2E_g, and $^2A_{1g}$) at the selected charge/discharge states.

For E-Bi_2Se_3 cathode (Supplementary Figure 19a), two characteristic signatures at 1360 and 1590 cm^{-1} corresponding to the D (disordered structure) and G (crystalline and graphitic structure) bands of acetylene black,[29] respectively, and other peaks are from the glass substrate. With the embedding of Zn^{2+}, the intensity of these characteristic peaks decreases, and the half-height width increases. With the release of Zn^{2+}, the intensity of the characteristic peak increases, and the half-

height width decreases (Supplementary Figure 19c).

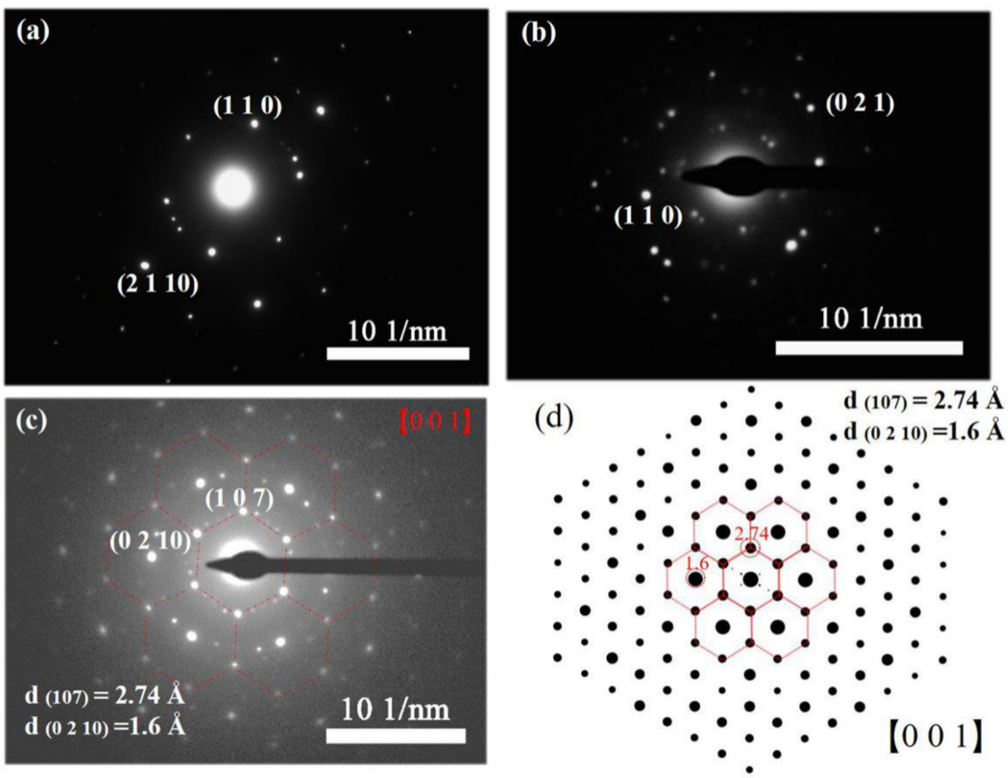

Supplementary Figure 20 (a, b, and c) SAED patterns of E-Bi_2Se_3 at a fully discharged state. SAED patterns in (a) and (b) show elongation of the d-spacing of the (110) plane. (d) Simulated electron diffraction pattern of $Zn_4Bi_2Se_3$ along [001] by the Mac Tempas software.[30]

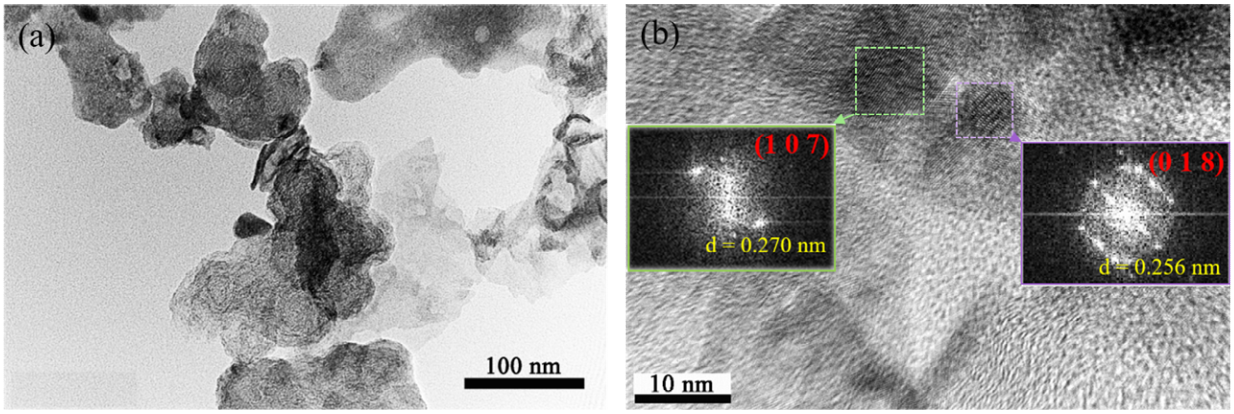

Supplementary Figure 21 TEM image (a) and high-resolution TEM (HRTEM) image (b) of E-Bi_2Se_3 at a full discharge state after 3 cycles. Insets in (b) display the corresponding fast Fourier transform (FFT) patterns.

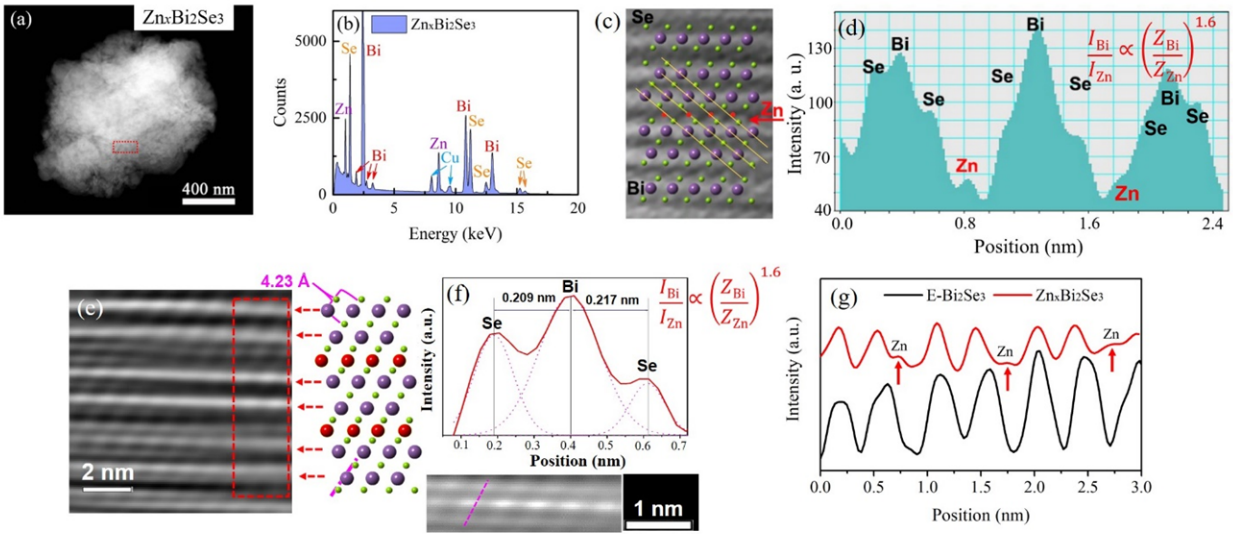

Supplementary Figure 22 (a) ADF-STEM image of $Zn_xBi_2Se_3$. The area highlighted with a red rectangular box is where the HAADF-STEM image (Figure 5h) is recorded. (b) EDS spectrum of $Zn_xBi_2Se_3$. We can see the clear signal of Zn to support the presence of Zn. (c) A magnified view with the superposition of the E-Bi_2Se_3 crystal structure. The yellow line shows (1 0 10)

crystallographic plane with the $[\overline{10}\ 0\ 1]$ crystallographic direction. (d) An averaged intensity profile from four traces of the (1 0 10) crystallographic plane in (c). The power law between (I_{Bi}/I_{Se}) and (Z_{Bi}/Z_{Se}) is analyzed with an averaging of three Se-Bi-Se peaks. (e) Magnified view of the red square area in Figure 5h. (f) Average line scan of the intensity profile (the peach dash line at the bottom of the HAADF image). (g) Comparison of the vertical line scan profiles of the HAADF images of E-Bi_2Se_3 in Figure 1g and ZnxBi_2Se_3 in Figure 5j.

Here the distance of Se to Bi atoms are 0.209 nm and 0.217 nm, respectively, and the measurement error is about 0.03 nm, so the Bi atom is apparently in the middle position between two Se atoms here (Supplementary Figure 22f). As we all known, the HAADF intensity I of one atom is proportional to its atom number Z of n power law $I=Z^n$ in the HAADF model.[31,32] In order to identify the power law n in this experimental and quantify the HAADF image of Zn^{2+} intercalating, As calculating the image contrast in intensity profile (I_{Bi}/I_{Se}) along the peach line of the HAADF image (Supplementary Figure 22f), an approximately proportional to the $(Z_{Bi}/Z_{Se})^{1.6}$ (Z is atomic number) confirms the ordered arrangement of Se and Bi atoms originating from the layered structure in the HAADF-STEM image (Figure 1g). Hence the power law n in this experiment has been determined to be 1.6.

As comparing the line scan profiles of the HAADF images of E-Bi_2Se_3 and ZnxBi_2Se_3 in Supplementary Figure 22g, we could find they are different apparently and some excess peaks site between two Se-Bi-Se columns in the ZnxBi_2Se_3 samples. In order to confirm the contribution of these peaks is from the insertion of Zn^{2+}, here we quantify the HAADF image by using the power law n=1.6. Firstly, we should point out that if these peaks are contributed from the distortion of Se, the intensity profiles should be very similar with the ones in Supplementary Figure 22f. Furthermore, the distance between the excess peak and the nearest Se-Bi-Se column neighbor is

about 0.290 nm, which is almost no atoms or intensity here in the HAADF image of $ZnxBi_2Se_3$ sample. As using the power law of *n*=1.6 to calculate the average atomic number of Z' in the excess peaks, we could find that the average atomic number *Z'* is about 28, which is smaller than the atomic numbers of Bi=83, Se=53, Zn=30 indicating an unfulfilled Zn^{2+} occupation inserted at the interval of two Se-Bi-Se columns (red spheres in Supplementary Figure 22e).

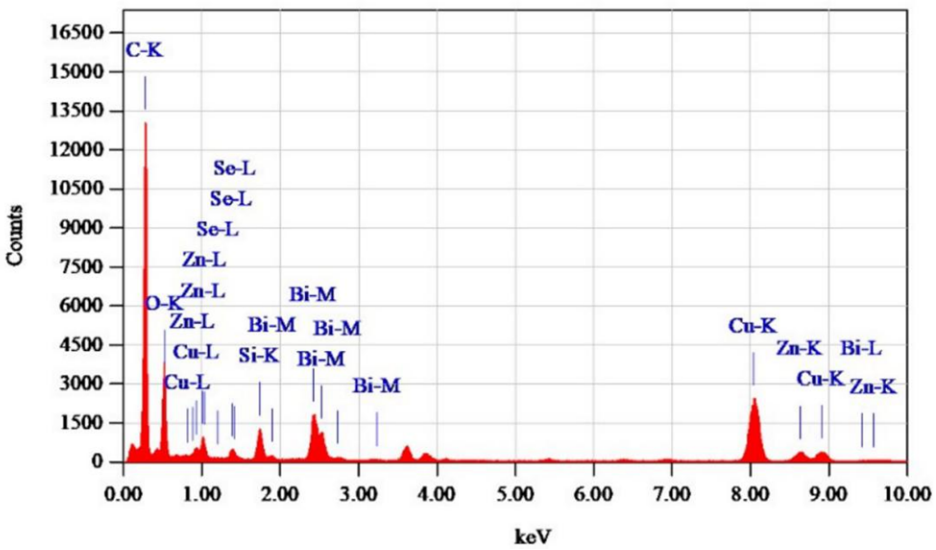

Supplementary Figure 23 Corresponding TEM-EDS spectrum of the fully discharged E-Bi_2Se_3.

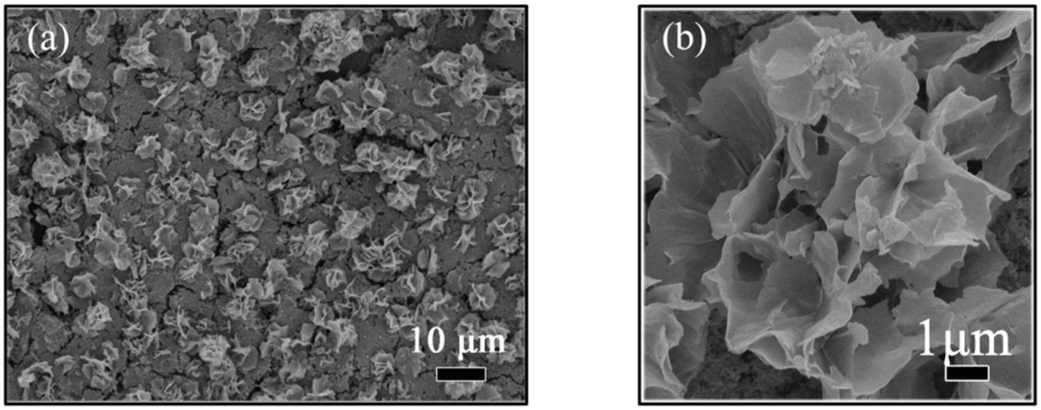

Supplementary Figure 24 SEM image of the E-Bi_2Se_3 electrode after 3 cycles under different magnification.

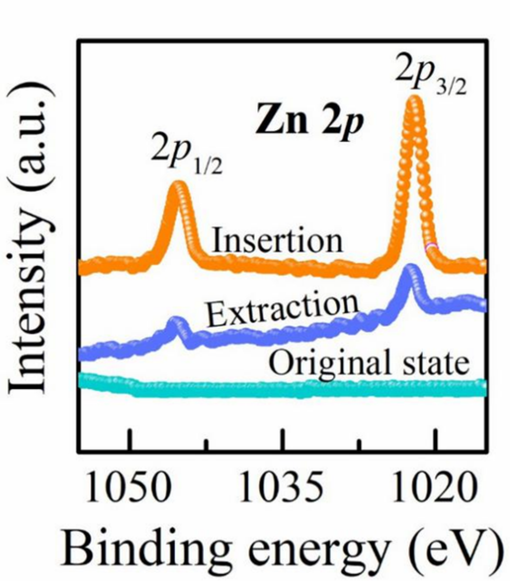

Supplementary Figure 25 High-resolution Zn 2*p* core level spectra of E-Bi_2Se_3 in initial state, charged to 2.3V, and discharged to 0.1V.

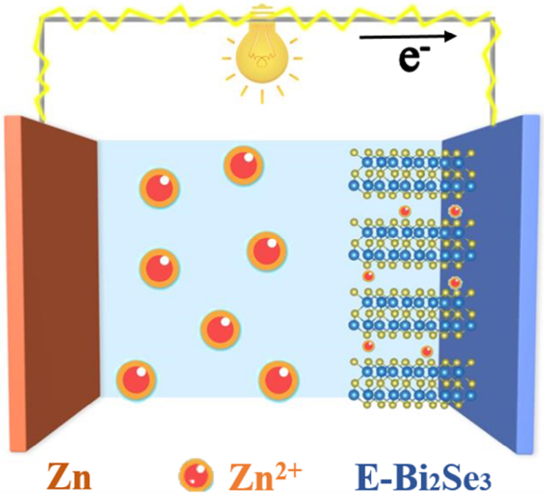

Supplementary Figure 26 Schematic diagram of the Zn||E-Bi_2Se_3 full cell. Clearly, the electrochemical reactions between the E-Bi_2Se_3 cathode and Zn anode can be listed as:

$$\text{Cathode: } Bi_2Se_3 + xZn^{2+} + 2xe^- \leftrightarrow Zn_xBi_2Se_3$$

$$\text{Anode: } xZn \leftrightarrow xZn^{2+} + 2xe^-$$

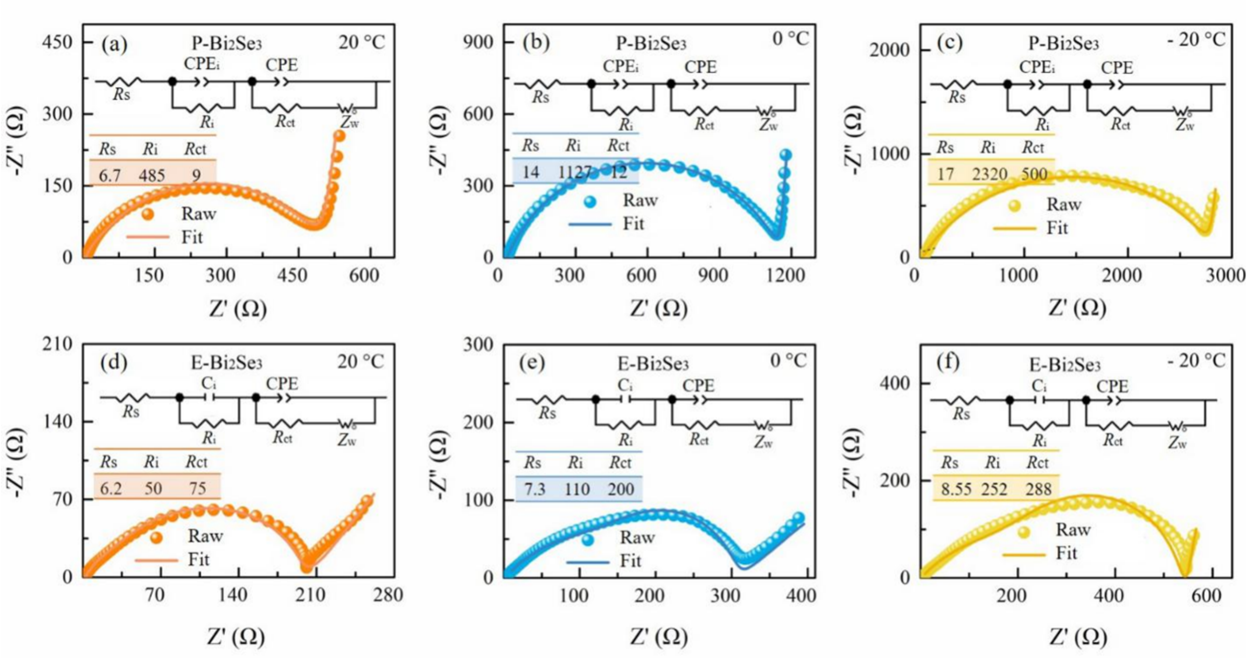

Supplementary Figure 27 EIS plots and fitted spectra of P-Bi_2Se_3 (a-c) and E-Bi_2Se_3 cells (d-f) at varying temperatures. The corresponding equivalent circuit models for the fitting and values of each resistance are listed in tables. Series resistance (R_s) includes the resistance of the electrolyte, separator and electrode materials. Interface resistance (R_i) and capacitance (C_i) between electrolyte and electrode are corresponding to the semicircle at high frequencies. Charge-transfer resistance (R_{ct}) is associated with the semicircle at medium frequency region. CPE represents constant phase element. Warburg impedance (Z_w) manifested into a straight sloping line at low-frequency end is related to the diffusion process, respectively.[33,34]

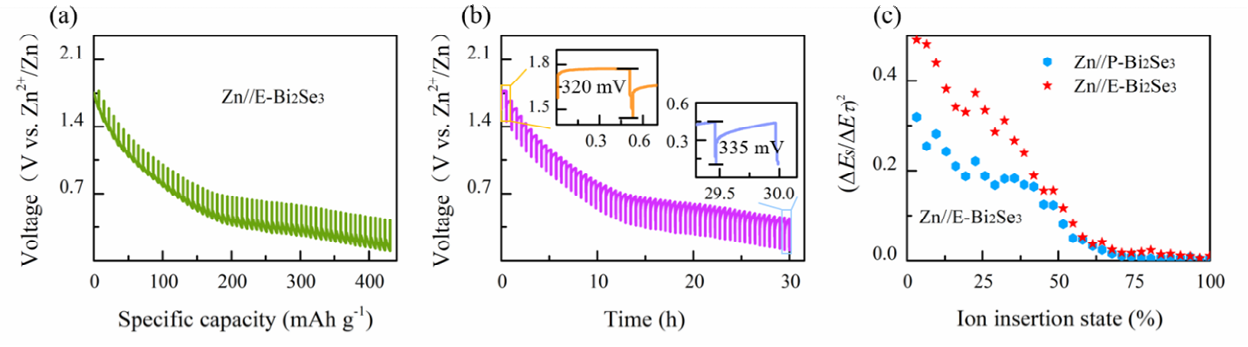

Supplementary Figure 28 The discharge galvanostatic intermittent titration technique (GITT) profiles of the Zn||E-Bi_2Se_3 (a, b) with insets in (b) showing total overvoltages during discharge at 0.2 A g^{-1} for 60 s followed by a 0.5 h rest. $(\Delta E_s/\Delta E_\tau)^2$calculated from GITT profiles of Zn||E-Bi_2Se_3 and Zn||P-Bi_2Se_3 cells as a function of ion insertion state. Here, the diffusion coefficient (D) of electrolyte ions can be calculated by the following equation[35]:

$$\mathrm{D} = \frac{4}{\pi\tau}\left(\frac{m_{\mathrm{B}}V_{\mathrm{M}}}{M_{\mathrm{B}}\mathrm{S}}\right)^2\left(\frac{\Delta E_{\mathrm{S}}}{\Delta E_{\tau}}\right) = \frac{4}{\pi\tau}\left(\frac{V_{\mathrm{M}}}{M_{\mathrm{B}}\left(\frac{S}{m_{\mathrm{B}}}\right)}\right)^2\left(\frac{\Delta E_{\mathrm{S}}}{\Delta E_{\tau}}\right)^2 = \frac{4}{\pi\tau}\left(\frac{V_{\mathrm{M}}}{M_{\mathrm{B}}(S_{\mathrm{BET}})}\right)^2\left(\frac{\Delta E_{\mathrm{S}}}{\Delta E_{\tau}}\right)^2$$

Where D has a linear relationship with $(\Delta E_{\mathrm{s}}/\Delta E_{\tau})^2$, ΔE_{s} and ΔE_τ are related to the variation of steady-state voltage, and the change of the voltage during titration for the corresponding step, respectively. τ, M_{B}, V_{M}, and S_{BET} are the duration of the current pulse, molecular mass, molar volume, and electrode-electrolyte interface based on the BET area, respectively. The diffusivity of Zn^{2+} in E-Bi_2Se_3 ranges from 4.3×10^{-10} to 5.1×10^{-12} cm^2 s^{-1} with an average of 1.5×10^{-10} cm^2 s^{-1}. Also, there are very little change in the total overpotentials in the initial state (320 mV) and the final state (335 mV) as shown in Supplementary Figure 10b.

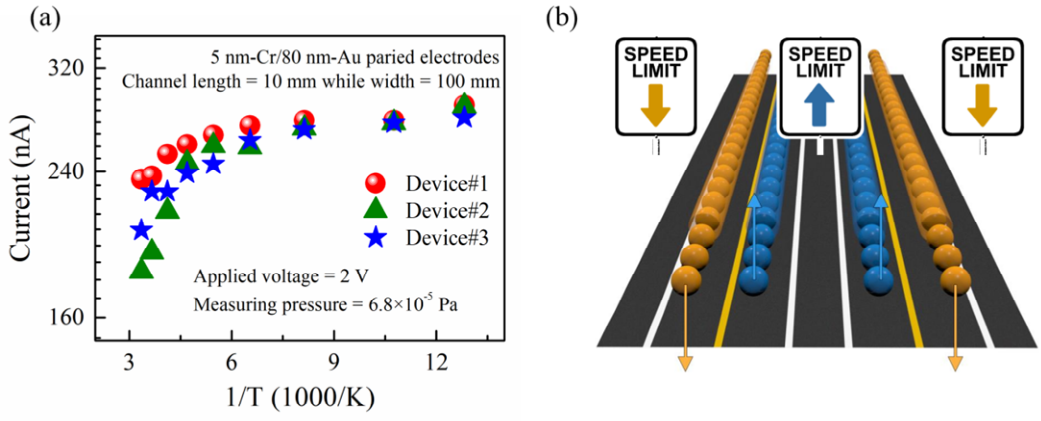

Supplementary Figure 29 (a) The temperature dependent current analysis on the single E-Bi_2Se_3 nanosheet (NS) device fabricated for the electrical transport experiment. (b) Two two-way highways with a restriction that these electrons (blue and yellow balls represent electrons that have different spin, and thus the diverse flow direction) cannot turn around and these currents stay constants beneficial from topological protection, which is based on Kramers theorem.[34] According to Kramers theorem, for one thing, these surface metal states allow the emergence of paired currents along the surface of the E-Bi_2Se_3, where these currents unstoppably flow in opposite direction restricted by the spin of electrons just like a two-way highway.[36] For another, the current maintains a constant, that is, E-Bi_2Se_3 are topologically protected.

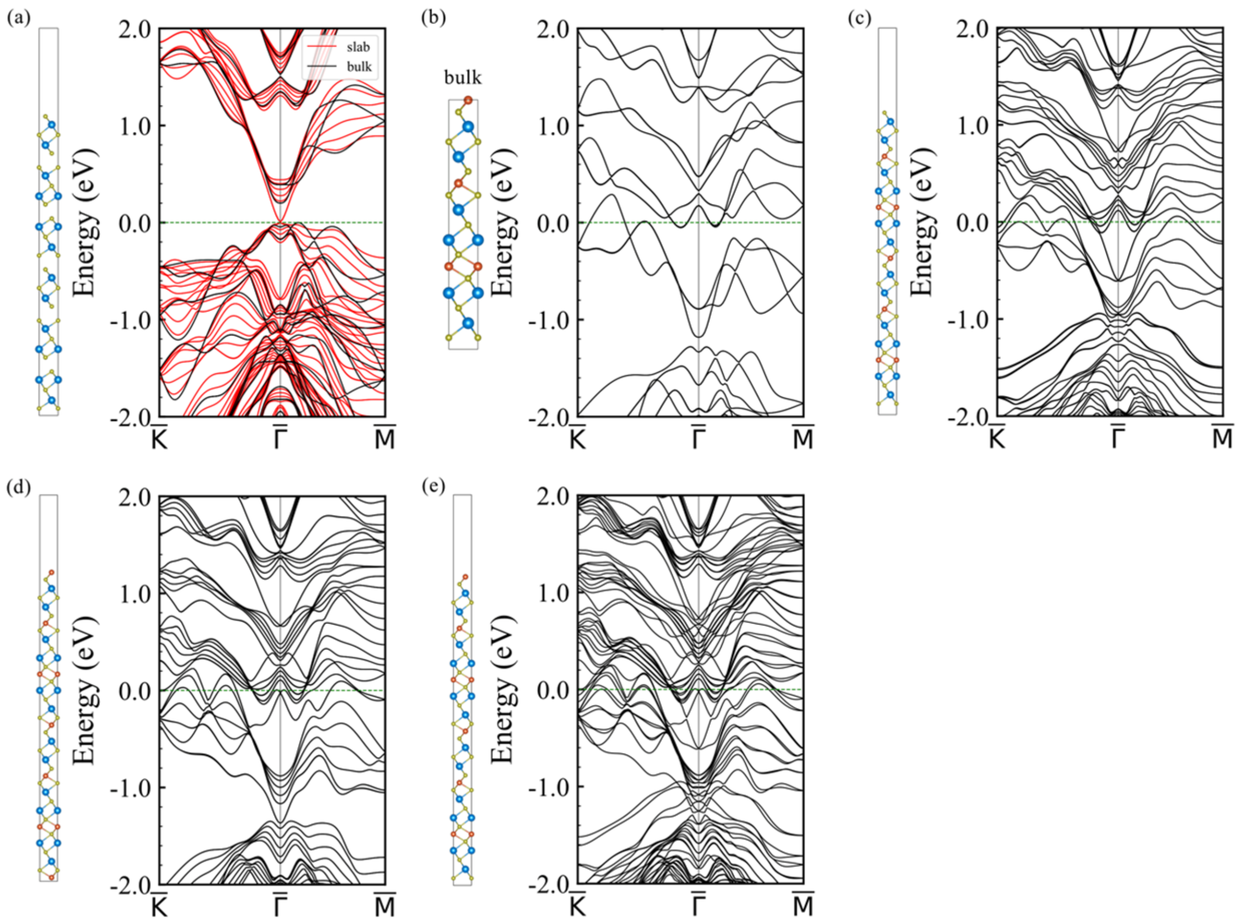

Supplementary Figure 30 Illustrations of atomic structure and SOC band structures of the 6-QL E-Bi_2Se_3 slab model and the case of charge neutral Zn atoms intercalated in E-Bi_2Se_3 interlayers ($ZnBi_2Se_3$). (a) SOC band structure 6-QL E-Bi_2Se_3 slab model. (b) SOC band structure of bulk $ZnBi_2Se_3$. The SOC band structures of 6-QL $ZnBi_2Se_3$ slab with Se-Se (c), Zn-Zn (d), and Zn-Se (e) termination.

The black lines in (a) corresponding to the bulk states show a band gap, while the red lines corresponding to the 6-QL slab and the gap is closed. It's obvious that the band connecting the conduction band and valence band is the topological surface state. For the Zn atoms intercalated systems, the Zn atoms are located in the center of Se octahedron retaining the original symmetry (b-e). From the bulk band structure, we can see the original gap of Bi_2Se_3 is pulled below 1.2 eV

of the Fermi level, and the conduction band passes through the Fermi level constituting the key signature of metallicity. Then slab model is adopted to calculate the surface state of the model, which is also metallic in nature. The original topological surface state of Bi_2Se_3 exists at about 1.0 eV under Fermi level. As it is too far away from Fermi level, this topological surface state contributes so little to conductivity that would not be shown remarkably in conductivity measurement. Therefore, the contribution of Zn atom (charge neutral) intercalation system to conductivity still comes from the metal state of itself.

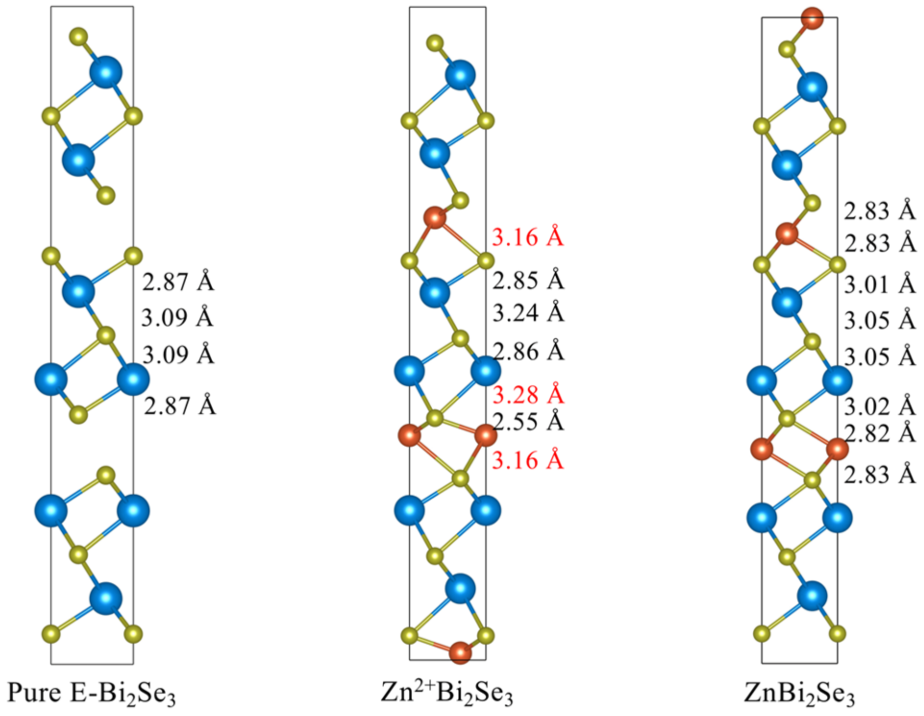

Supplementary Figure 31 Structural information of pure E-Bi_2Se_3, Zn^{2+} intercalated in three interlayers ($Zn^{2+}Bi_2Se_3$) and neutral Zn atoms intercalated in E-Bi_2Se_3 ($ZnBi_2Se_3$). The figures are the bond length of atoms adjacent to the numbers.

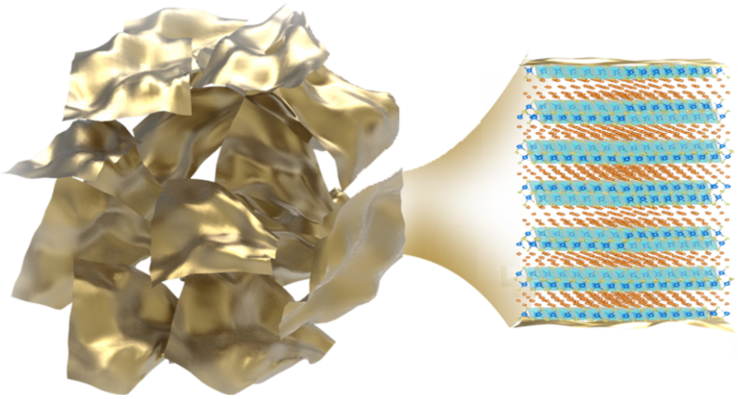

Supplementary Figure 32 Schematic drawing of the $Zn_xBi_2Se_3$ with enhanced metal-like conductivity coming from trivial metal surface state

Supplementary Table 1 ICP-AES and ICP-MS results for prepared E-Bi_2Se_3

Element	Concentration (ppm)	Molar ratio*
Li	9.68	0.00
Bi	323950	1.00
Se	181420	1.48

*Molar ratio normalized to Bi

Supplementary Table 2 Crystal data and structure refinement conditions for the P-Bi_2Se_3 and E-Bi_2Se_3

Compound	P-Bi_2Se_3	E-Bi_2Se_3	$Zn_4Bi_2Se_3$
cryst syst	rhombohedral	rhombohedral	rhombohedral
a (Å)	4.13774	4.14332	4.18684
b (Å)	4.13774	4.14332	4.18684
c (Å)	28.6575	28.6740	29.6090
V (Å^3)	424.9084	426.3007	449.49
space group	$R\bar{3}m$	$R\bar{3}m$	$R\bar{3}m$
reduced χ^2	2.395	8.15	8.86
R_{wp} (%)	5.83	3.84	6.32
R_p (%)	4.32	2.96	4.65

Supplementary Table 3 Fitted impendence parameters of Zn||P-Bi_2Se_3 and Zn||E-Bi_2Se_3 cells in the original state and after cycles. R_s, R_{ct}, and CPE represent series resistance, charge-transfer resistance and constant phase element, respectively. R_i and Z_w indicate interface resistance between electrolyte and electrode, and Warburg impedance, respectively.

	Zn||P-Bi_2Se_3		Zn||E-Bi_2Se_3					
	Pristine cell		Pristine cell		1300 cycles		2300 cycles	
	Value (Ω)	Error (%)	Value	Error	Value	Error	Value	Error
R_s	10.85	0.54	5.6	0.3	5.79	0.59	6.38	0.8
R_i	---	---	---	---	19.03	2.17	40	1.35
R_{ct}	1860	6.73	583.1	5.32	88.4	1.24	90.6	3.97
Z_w	660.9	3.41	243.9	4.16	239.9	5.63	890	6.05

Equivalent circuit 1

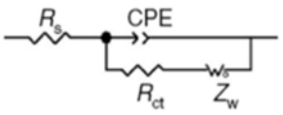

2

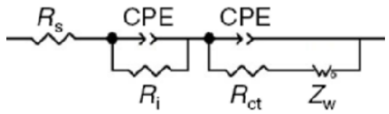

References

1 Ren, L. et al. Large-scale production of ultrathin topological insulator bismuth telluride nanosheets by a hydrothermal intercalation and exfoliation route. J. Mater. Chem. 22, 4921 (2012).

2 Egerton, R. F. Control of radiation damage in the TEM. Ultramicroscopy. 127, 100-108 (2013).

3 Nian, Q. *et al*. Aqueous batteries operated at -50 °C. *Angew. Chem. Int. Ed.* **131**, 17150-17155 (2019).

4 Dong, X. *et al*. Organic batteries operated at -70 °C. *Joule* **2**, 902-913 (2018).

5 Yang, Y. *et al.* Fluorinated carboxylate ester-based electrolyte for Li ion batteries operated at low temperature. *Chem. Commun.* **56**, 9640-9643 (2020).

6 Eftekhari, A. Supercapacitors utilising ionic liquids. *Energy Stor. Mater.* **9**, 47-69 (2017).

7 Liu, H. *et al.* A large capacity of LiV_3O_8 cathode material for rechargeable lithium-based batteries. *Electrochim. Acta* **56**, 1392-1398 (2011).

8 Mo, F. *et al.* A flexible rechargeable aqueous zinc manganese-dioxide battery working at -20 °C. *Energy Environ. Sci.* **12**, 706-715 (2019).

9 Chen, M. *et al.* Realizing an all-round hydrogel electrolyte toward environmentally adaptive dendrite free aqueous Zn-MnO_2 Batteries. *Adv. Mater.* **31**, 1901521 (2019).

10 Peng, H. *et al.* Aharonov-Bohm interference in topological insulator nanoribbons. *Nat. Mater.* **9**, 225-229 (2010).

11 Hong, S. S. *et al.* Ultrathin topological insulator Bi_2Se_3 nanoribbons exfoliated by atomic force microscopy. *Nano Lett.* **10**, 3118-3122 (2010).
12 Huang, Y. *et al.* A self-healable and highly stretchable supercapacitor based on a dual crosslinked polyelectrolyte. *Nat Commun* **6**, 10310 (2015).
13 Xu, C. J. *et al.* Energetic zinc ion chemistry: the rechargeable zinc ion battery. *Angew. Chem. Int. Ed.* **51**, 933-935 (2012).
14 Alfaruqi, M. H. *et al.* A layered δ-MnO_2 nanoflake cathode with high zinc-storage capacities for eco-friendly battery applications. *Electrochem. Commun*. **60**, 121-125 (2015).
15 Lee, J. *et al.* Todorokite-type MnO_2 as a zinc-ion intercalating material. *Electrochim. Acta* **112**, 138-143 (2013).
16 Zhang, N. *et al.* Cation-deficient spinel $ZnMn_2O_4$ cathode in $Zn(CF_3SO_3)_2$ electrolyte for rechargeable aqueous Zn-ion battery. *J. Am. Chem. Soc.* **138**, 12894-12901 (2016).
17 Zhang, L. *et al.* Towards high-voltage aqueous metal-ion batteries beyond 1.5 V: the zinc/zinc hexacyanoferrate system. *Adv. Energy Mater.* **5**, 1400930 (2015).
18 Trocoli, R. *et al.* An aqueous zinc-ion battery based on copper hexacyanoferrate. *ChemSusChem* **8**, 481-485 (2015).
19 He, P. *et al.* Layered VS_2 nanosheet ‐ based aqueous Zn ion battery cathode. *Adv. Energy Mater.* **7**, 1601920 (2017).
20 Xia, C. *et al*. Rechargeable aqueous zinc-ion battery based on porous framework zinc pyrovanadate intercalation cathode. *Adv. Mater.* **30**, 1705580 (2018).
21 Kundu, D. *et al*. A high-capacity and long-life aqueous rechargeable zinc battery using a metal oxide intercalation cathode. *Nat. Energy* **1**, 16119 (2016).
22 Liu, Z. *et al*. Bio-degradable zinc-ion battery based on a prussian blue analogue cathode and a bio-ionic liquid-based electrolyte. *J. Solid State Electrochem*. **21**, 2021-2027 (2017).
23 Zhang, B. *et al*. An aqueous rechargeable battery based on zinc anode and $Na_{0.95}MnO_2$. *Chem. Commun.* **50**, 1209-1211 (2014).
24 Pang, Q. *et al*. $H_2V_3O_8$ nanowire/graphene electrodes for aqueous rechargeable zinc ion batteries with high rate capability and large capacity. *Adv. Energy Mater.* **8**, 1800144 (2018).
25 Li, G. *et al*. Hybrid aqueous battery based on $Na_3V_2(PO_4)_3$/C cathode and zinc anode for potential large-scale energy storage. *J. Power Sources* **308**, 52-57 (2016).
26 Aurbach, D. et al. Review of selected electrode-solution interactions which determine the performance of Li and Li ion batteries. J. Power Sources 89, 206-218 (2000).
27 Zhang, N. *et al.* Rechargeable aqueous zinc-manganese dioxide batteries with high energy and power densities. *Nat Commun* **8**, 405 (2017).
28 Guo, S. *et al.* Environmentally stable interface of layered oxide cathodes for sodium-ion batteries. *Nat Commun* **8**, 135 (2017).
29 Hu, H. Y. *et al*. Enhancing the performance of motive power lead-acid batteries by high surface area carbon black additives. *Appl. Sci.* **9**, 186 (2019).
30 O'Keefe, M. A. *et al*. MacTempas, V1.70 and Crystalkit V1.77, HRTEM Image Analysis; Lawrence Berkley National Laboratory: Berkeley, CA, 1987.
31 Yamashita, S. *et al.*, Atomic number dependence of *Z* contrast in scanning transmission electron microscopy. *Sci Rep* **8,** 12325 (2018).
32 Nellist, P.D. *et al.* Incoherent imaging using dynamically scattered coherent electrons. *Ultramicroscopy* **78**, 111-124 (1999).
33 Zhang, S. S. *et al.* Electrochemical impedance study on the low temperature of Li-ion batteries. *Electrochim. Acta* **49**, 1057-1061 (2004).
34 Li, X. *et al.* Hydrated hybrid vanadium oxide nanowires as the superior cathode for aqueous Zn battery. *Mater. Today Energy* **14**, 100361 (2019).
35 Lee, B. *et al.* Elucidating the intercalation mechanism of zinc ions into alpha-MnO_2 for rechargeable zinc batteries. *Chem. Commun. (Camb)* **51**, 9265-9268 (2015).

36 Zhang, H. *et al.* Topological insulators in Bi_2Se_3, Bi_2Te_3 and Sb_2Te_3 with a single Dirac cone on the surface. *Nat. Phys.* **5**, 438-442 (2009).
