# Supplementary material for: Few-layer bismuth selenide cathode for low-temperature quasi-solid-state aqueous zinc metal batteries
Source: Nat Commun. 2022 Feb 8;13:752. doi: 10.1038/s41467-022-28380-y (PMC8825835; doi:10.1038/s41467-022-28380-y)
Supplement: Supplementary file 3 — Reporting Summary [file 41467_2022_28380_MOESM3_ESM.pdf]

nature portfolio

Corresponding author(s): ZHI Chunyi

Last updated by author(s): 16/12/21

Reporting Summary

Nature Portfolio wishes to improve the reproducibility of the work that we publish. This form provides structure for consistency and transparency in reporting. For further information on Nature Portfolio policies, see our Editorial Policies and the Editorial Policy Checklist.

Statistics

For all statistical analyses, confirm that the following items are present in the figure legend, table legend, main text, or Methods section.

n/a	Confirmed	
☒	☐	The exact sample size (*n*) for each experimental group/condition, given as a discrete number and unit of measurement
☒	☐	A statement on whether measurements were taken from distinct samples or whether the same sample was measured repeatedly
☒	☐	The statistical test(s) used AND whether they are one- or two-sided<br>*Only common tests should be described solely by name; describe more complex techniques in the Methods section.*
☒	☐	A description of all covariates tested
☒	☐	A description of any assumptions or corrections, such as tests of normality and adjustment for multiple comparisons
☒	☐	A full description of the statistical parameters including central tendency (e.g. means) or other basic estimates (e.g. regression coefficient) AND variation (e.g. standard deviation) or associated estimates of uncertainty (e.g. confidence intervals)
☒	☐	For null hypothesis testing, the test statistic (e.g. *F*, *t*, *r*) with confidence intervals, effect sizes, degrees of freedom and *P* value noted<br>*Give P values as exact values whenever suitable.*
☒	☐	For Bayesian analysis, information on the choice of priors and Markov chain Monte Carlo settings
☒	☐	For hierarchical and complex designs, identification of the appropriate level for tests and full reporting of outcomes
☒	☐	Estimates of effect sizes (e.g. Cohen's *d*, Pearson's *r*), indicating how they were calculated

Our web collection on statistics for biologists contains articles on many of the points above.

Software and code

Policy information about availability of computer code

Data collection	Structural characterizations were conducted on Bruker D2 Phaser diffractometer, Hitachi S4700, WITec alpha300 access spectrometer; Electrochemical characterizations were conducted on CHI760E electrochemical workstation, LAND CT2001A battery testing system. Density functional theory (DFT) calculations were performed on Vienna ab-initio Simulation Package (VASP).
Data analysis	origin

For manuscripts utilizing custom algorithms or software that are central to the research but not yet described in published literature, software must be made available to editors and reviewers. We strongly encourage code deposition in a community repository (e.g. GitHub). See the Nature Portfolio guidelines for submitting code & software for further information.

Data

Policy information about availability of data

All manuscripts must include a data availability statement. This statement should provide the following information, where applicable:

- Accession codes, unique identifiers, or web links for publicly available datasets
- A description of any restrictions on data availability
- For clinical datasets or third party data, please ensure that the statement adheres to our policy

Provide your data availability statement here.

Field-specific reporting

Please select the one below that is the best fit for your research. If you are not sure, read the appropriate sections before making your selection.

☐ Life sciences ☐ Behavioural & social sciences ☒ Ecological, evolutionary & environmental sciences

For a reference copy of the document with all sections, see nature.com/documents/nr-reporting-summary-flat.pdf

Ecological, evolutionary & environmental sciences study design

All studies must disclose on these points even when the disclosure is negative.

Study description	Few-layer bismuth selenide cathode for low-temperature quasi-solid-state aqueous zinc metal batteries
Research sample	Bi2Se3, lithium bis-trifluoromethanesulfonimide (LiTFSI), zinc bis(trifluoromethanesulfonyl)imide (Zn(TFSI)2)
Sampling strategy	The electrode was prepared by mixing Bi2Se3 with ketjenblack and polyvinylidene fluoride in N-methyl-2-pyrrolidinone solvents to form a homogeneous slurry, which was then coated on a carbon cloth and dried, respectively. The electrolyte was 1m Zn(TFSI)2 and 21m LiTFSI. The full cell was assembled with Zn plate as anode and Bi2Se3 as cathode.
Data collection	Structural characterizations were conducted on Bruker D2 Phaser diffractometer, Hitachi S4700, WITec alpha300 access spectrometer; Electrochemical characterizations were conducted on CHI760E electrochemical workstation, LAND CT2001A battery testing system. Density functional theory (DFT) calculations were performed on Vienna ab-initio Simulation Package (VASP).
Timing and spatial scale	Mar. 2021-Dec. 2021
Data exclusions	No data were excluded.
Reproducibility	All attempts to repeat the experiment were successful.
Randomization	Not applicable.
Blinding	Not applicable.

Did the study involve field work? ☐ Yes ☒ No

Reporting for specific materials, systems and methods

We require information from authors about some types of materials, experimental systems and methods used in many studies. Here, indicate whether each material, system or method listed is relevant to your study. If you are not sure if a list item applies to your research, read the appropriate section before selecting a response.

Materials & experimental systems

n/a	Involved in the study	
☒	☐	Antibodies
☒	☐	Eukaryotic cell lines
☒	☐	Palaeontology and archaeology
☒	☐	Animals and other organisms
☒	☐	Human research participants
☒	☐	Clinical data
☒	☐	Dual use research of concern

Methods

n/a	Involved in the study	
☒	☐	ChIP-seq
☒	☐	Flow cytometry
☒	☐	MRI-based neuroimaging
